# SLC39-driven zinc influx orchestrates pleiotropic tumor–immune system cross-talk to establish immune suppression in colorectal cancer

**DOI:** 10.1126/sciadv.aeh2362

**Published:** 2026-10-07

**Authors:** Seok June Hong, Seheum Park, Sunghoon Kim, Young Il Park, Jun Seok Lee, Mikyung Kang, Jaewoong Lee, Martin Hemberg, Sung Eun Kim, Kwoneel Kim

**Affiliations:** ^1^ Department of Biomedical and Pharmaceutical Sciences, Kyung Hee University, Seoul, Republic of Korea.; ^2^ School of Biosystems and Biomedical Sciences, College of Health Sciences, Korea University, Seoul 02841, Republic of Korea.; ^3^ Department of Integrated Biomedical and Life Sciences, College of Health Sciences, Korea University, Seoul 02841, Republic of Korea.; ^4^ L-HOPE Program for Community-Based Total Learning Health Systems, Korea University, Seoul 02841, Republic of Korea.; ^5^ Department of Health and Safety Convergence Science, College of Health Sciences, Korea University, Seoul 02841, Republic of Korea.; ^6^ School of Health and Environmental Science, College of Health Science, Korea University, Seoul 02841, Republic of Korea.; ^7^ Department of Health and Safety Convergence Science, College of Health Science, Korea University, Seoul 02841, Republic of Korea.; ^8^ Biomedical Research Center, Korea University Guro Hospital, Seoul 08308, Republic of Korea.; ^9^ Interdisciplinary Program in Precision Public Health, Korea University, Seoul 02841, Republic of Korea.; ^10^ Center of Molecular and Cellular Oncology, Yale University, New Haven, CT 06511, USA.; ^11^ Gene Lay Institute of Immunology and Inflammation, Brigham and Women’s Hospital, Harvard Medical School and Massachusetts General Hospital, Boston, MA 02115, USA.; ^12^ Department of Biology, Kyung Hee University, Seoul, Republic of Korea.

## Abstract

Tumor metabolic programs shape immune evasion; however, the mechanism by which nutrient-transport systems rewire tumor-immune cell interactions remains unknown. We integrated bulk multi-omics, single-cell transcriptomics, spatial profiling, and functional assays for colorectal cancer–associated zinc-driven immunosuppressive circuit identification. Multi-omics analysis of 258 patients revealed three solute carrier transporter-centered archetypes, with an SLC39-enriched subtype aligned with consensus molecular subtype 2–like immune-desert tumors. SLC39-mediated zinc influx activated CDX2, which engaged a distal enhancer to induce tumor cell-specific CD24 expression. CD24^+^ tumor cells formed spatial niches with SIGLEC10-expressing monocyte-derived macrophages, dendritic cells, and resident macrophages, activating pleiotropic programs that suppress phagocytosis and remodel adhesion. SLC39 overexpression increased intracellular zinc and CD24 levels and reduced macrophage-mediated engulfment, whereas CD24 blockade restored phagocytosis. The zinc-CDX2-CD24-SIGLEC10 axis defined a terminal cancer cell state associated with poor survival and immune checkpoint blockade resistance. A seven-gene signature predicted immunotherapy responses across five colorectal cancer cohorts, identifying SLC39-driven zinc influx as a metabolic checkpoint governing immune exclusion.

## INTRODUCTION

Tumor cells exhibit heightened metabolic demands and reprogram nutrient acquisition through solute carrier (SLC) transporters to sustain uncontrolled proliferation, survival, and metastasis. Members of the SLC family have emerged as pivotal mediators of metabolic reprogramming, facilitating the import of essential nutrients and export of metabolic waste, thereby coupling metabolic flux to oncogenic signaling (*1*, *2*). The expression of SLC2A1 (GLUT1), a key glucose transporter, is up-regulated in many cancers to support aerobic glycolysis, whereas SLC7A5 (LAT1) enables the uptake of large neutral amino acids such as leucine, sustaining protein synthesis and mTORC1 activation (*2*). The therapeutic inhibition of LAT1 has shown efficacy in preclinical models by restricting amino acid availability and suppressing tumor progression. Similarly, SLC7A11 (xCT) imports cystine to fuel glutathione biosynthesis, enhance antioxidant defense, and protect cancer cells from ferroptosis (*3*). These findings underscore the role of SLC transporters as central mediators of metabolic reprogramming and as potential therapeutic targets in oncology (*4*).

Although metabolic rewiring is a hallmark of cancer, the extent to which SLC transporters orchestrate interactions between tumor cells and the tumor microenvironment (TME) remains poorly defined. Accumulating evidence suggests that metabolic by-products generated through the Warburg effect, such as lactate, can acidify the TME and impair immune cell activity, thereby facilitating immune escape (*5*). However, most studies have emphasized tumor-intrinsic metabolic programs, while neglecting the reciprocal influence of stromal and immune components (*6*). As such, current frameworks do not adequately capture the interplay between metabolic adaptation and immune modulation. This disconnect is particularly critical when considering that metabolic competition and nutrient availability in the TME can shape both tumor behavior and the therapeutic response. The absence of integrated systems-level studies that dissect these interactions has limited our capacity to identify context-specific metabolic vulnerabilities and design effective metabolism-targeted therapies.

Colorectal cancer (CRC) provides a well-established framework for dissecting the interplay between tumor metabolism and the immune microenvironment through consensus molecular subtypes (CMS). CMS1 tumors are characterized by microsatellite instability, hypermutations, and prominent immune infiltration, whereas CMS2 tumors display canonical WNT and MYC signaling accompanied by an immune-desert phenotype. CMS3 tumors exhibit marked metabolic dysregulation, and CMS4 tumors are characterized by stromal activation, mesenchymal features, and poor clinical outcomes (*7*), together accounting for approximately 16, 32, 17, and 35% of CRC cases, respectively (*8*). These subtype-resolved architectures underscore the heterogeneity of CRC and highlight the need to identify molecular regulators that couple metabolic programs with the immune context. Despite the extensive molecular characterization of CMS subtypes, the contribution of SLC transporters to CRC subtype identity and TME modulation remains unclear. Zinc is an essential micronutrient required for a wide range of enzymatic, transcriptional, and metabolic processes, and the tight regulation of intracellular zinc homeostasis is critical for cellular functions (*9*, *10*). Zinc flux is primarily controlled by the SLC39 family of transporters, which mediates zinc influx into the cytoplasm, and by the SLC30 family, which facilitates zinc efflux (*11*–*14*). Although individual zinc transporters have been linked to tumor growth and progression in CRC (*15*, *16*), systematic studies addressing their functional roles and downstream signaling consequences across molecular subtypes are lacking. In particular, the intersection of zinc transporter activity with immune regulation within the TME remains largely unexplored. Here, we propose that SLC39-mediated zinc influx constitutes a central metabolic cue that drives subtype-specific transcriptional reprogramming in CRC, promoting immune checkpoint activation and inhibiting antitumor immune responses. We hypothesized that differential zinc transporter expression defines metabolically regulated immunosuppressive states across CRC subtypes. By integrating bulk and single-cell multi-omics with spatial profiling approaches, the aim of our study was to delineate the SLC-TME axis as a mechanistic link between metabolic inputs and immune modulation, thereby uncovering actionable vulnerabilities within distinct CRC subtypes.

## RESULTS

### SLC transporter programs define metabolic-immune archetypes in patients with CRC

SLC transporters are pivotal mediators of the metabolite flux that sculpts the TME (*17*). To elucidate the regulatory architecture of SLC transporters in CRC, we conducted integrative multi-omics profiling encompassing more than 300 SLC transporter genes and 500 cancer-driver genes across 258 The Cancer Genome Atlas Program-colon adenocarcinoma (TCGA-COAD) patient samples (Fig. 1A). The multi-omics dataset included transcriptomic alterations, somatic mutations, copy number variations (CNVs), and DNA methylation profiles, enabling a comprehensive assessment of SLC transporter-associated regulatory programs. We then used Multi-Omics Factor Analysis (MOFA) (*18*) to extract latent molecular factors from the integrated multi-omics data and selected the factor combination that best captured the archetypal structure using the ParetoTI *t*-ratio metric (fig. S1A). Functional annotation of the selected factors revealed strong associations with metabolic and immune-related pathways (Fig. 1B). The projection of patients onto this factor space identified three distinct SLC-centered archetypes that were subsequently characterized by SLC transporter enrichment, pathway activity, tumor microenvironmental signatures, and CMS associations (Fig. 1, C and D). Among these, archetype 1 exhibited the notable enrichment of SLC39 family members at the gene expression level (*SLC39A2*, *SLC39A4*, *SLC39A5*, and *SLC39A10* ranked in the top 5%, fig. S1B), whereas the SLC features associated with archetypes 2 and 3 were primarily linked to DNA methylation, indicating archetype-specific differences in the dominant omics modality. The enrichment of SLC39 expression correlated with the activation of zinc-related pathways. In line with prior studies implicating the SLC39 family in zinc import (*13*), zinc influx transporters were more highly expressed than their SLC30 family-mediated efflux counterparts (fig. S1C). Patients within this archetype were predominantly aligned with the CMS2 molecular subtype, which is characterized by WNT activation, microsatellite stability (MSS), and an immune-desert phenotype (*19*). In line with these features, this archetype showed elevated activity of the WNT- and MSS-associated pathways, while displaying the depletion of key infiltrating immune cells, as inferred from the transcriptomic enrichment analysis. Notably, the association between archetype 1 and an immunorepressive state remained significant, independent of the MSS status (fig. S1D), indicating that immune exclusion is a defining feature of this subgroup, rather than a secondary consequence of genomic stability. These findings define archetype 1 as a CMS2-like, immune-suppressed CRC subtype characterized by SLC39 overexpression and enhanced zinc transport, establishing a molecular framework linking SLC-mediated metabolic influx to immune-desert tumor states.

**Fig. 1.**
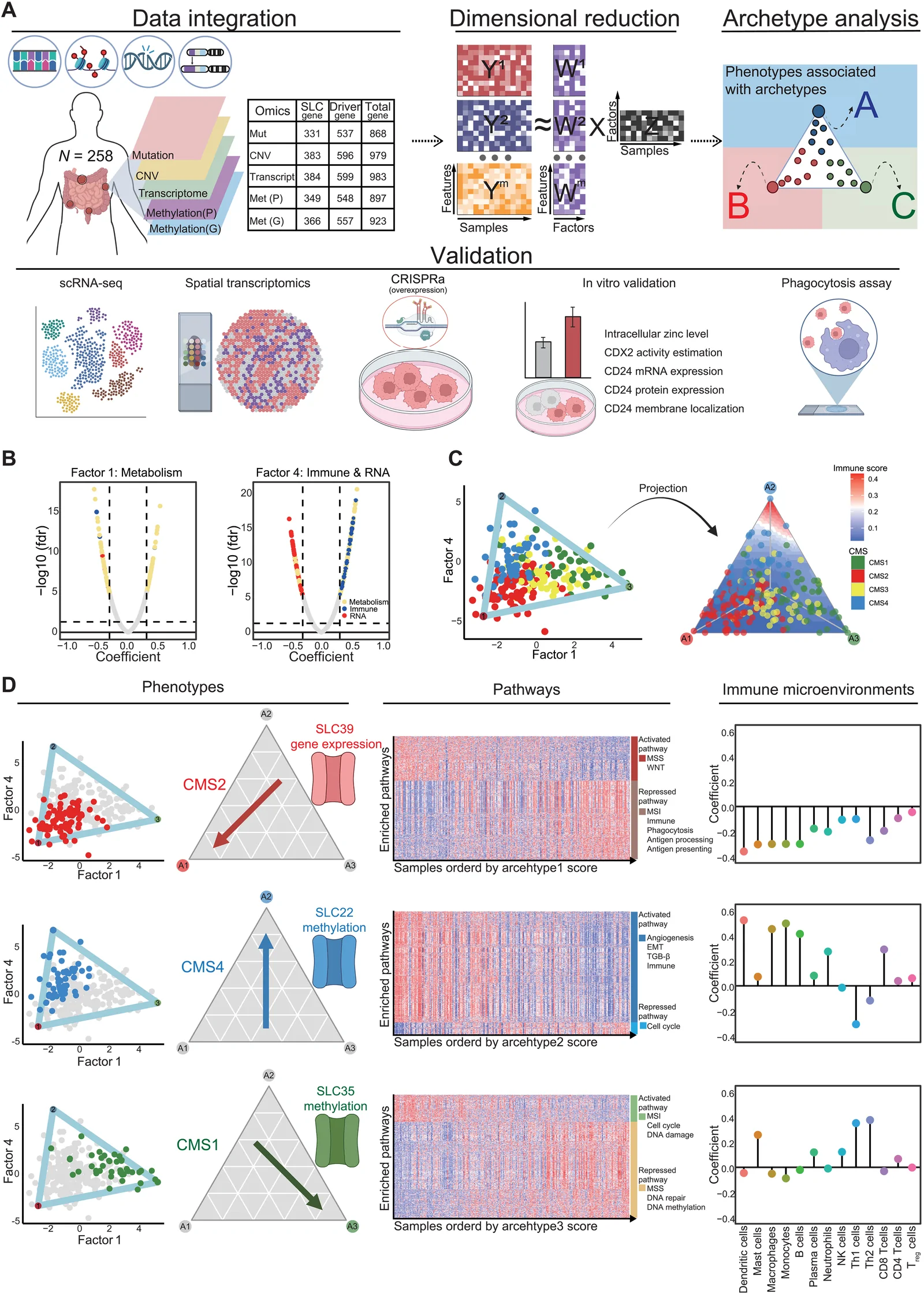
Study design and SLC-centered archetypes in CRC. (**A**) Schematic representation of the study design. Multi-omics data from 258 patients with CRC in the TCGA cohort were collected and filtered to only include SLC genes and known cancer driver genes. (**B**) Latent factor 1 was predominantly associated with metabolic pathways and latent factor 4 was enriched for immune-related and RNA processing pathways. Each dot represents the correlation coefficient for a pathway obtained from Pearson correlation analysis between latent factor values and pathway activity scores following sample-based pathway analysis. (**C**) Sample projection in latent factor space. Samples were plotted along metabolic (factor 1) and immune and RNA (factor 4) axes and mapped onto a ternary plot indicating relative distances to the three inferred archetypes. Points are colored by CMS, with background shading reflecting immune infiltration scores. (**D**) Overview of phenotypes, pathways, and tumor immune microenvironment identified in each archetype. Left: Samples with specific CMSs were mapped onto the latent factor space and arrows indicate representative SLC gene families enriched in each archetype. Middle: Expression heatmaps of representative pathways. Rows indicate the pathways that are either positively or negatively enriched, while columns indicate the samples ordered by increasing archetype scores. Colored annotations on the right highlight representative pathways previously reported to be associated with specific CMSs. Right: Correlation between archetype scores and immune cell enrichments inferred by xCell analysis. Created in BioRender. Lab, S. (2026) https://BioRender.com/ebz4tp7.

### SLC39-mediated zinc influx promotes CDX2-dependent CD24 enhancer activation

To elucidate the molecular mechanisms underlying SLC39 family overexpression and zinc transport activation in archetype 1, we identified the key signaling molecules associated with this pathway (Fig. 2A, top). An analysis of cancer drivers relevant to archetype 1 revealed CDX2 as a top-ranked transcription factor (TF), along with the SLC39 family (fig. S1, E and F). CDX2 plays critical roles in gut development and homeostasis (*20*). Notably, both CDX2 expression and the inferred regulatory activity were positively correlated with SLC39-mediated zinc influx and an archetype 1 status (fig. S2A). To prioritize immune-relevant CDX2 downstream effectors associated with archetype 1, we curated 115 known immunomodulatory genes (*21*, *22*) and correlated their expression levels with archetype 1 scores. The analysis identified CD24 as the most strongly associated immunomodulatory candidate (fig. S2B), which is consistent with its reported function in mediating immune-repressive signaling (*23*) and aligns with the immune-desert characteristics of archetype 1. Across TCGA and Clinical Proteomic Tumor Analysis Consortium (CPTAC) CRC cohorts, the expression of SLC39 transporters, CDX2, and CD24 demonstrated broad positive correlations with zinc pathway activity (Fig. 2A), supporting a zinc-driven transcriptional cascade linking SLC39 influx to CDX2-dependent CD24 activation. This regulatory cascade was further supported by COMPASS-based metabolic flux modeling (*24*), which quantified zinc-associated reaction-level flux states beyond conventional gene set scoring and independently recapitulated the SLC39-CDX2-CD24 signaling axis (fig. S2C). Although CDX2 is an intestinal lineage factor (*20*), models incorporating both zinc influx and CDX2 activity explained CD24 expression better than those incorporating CDX2 activity alone (fig. S2D), and mediation analysis indicated that zinc influx induced CD24 expression primarily through CDX2 activity (fig. S2E). To investigate the genomic basis of SLC39 overexpression, we analyzed CNV and identified a positive correlation between SLC39 expression and CNV in both the TCGA and CPTAC cohorts (Fig. 2B). In addition, CNV inference from the single-cell transcriptomic data of 21,698 CRC cells (*25*) using the inferCNV algorithm (*26*) confirmed genomic amplification associated with SLC39 family overexpression. Given that CDX2 functions as a transcriptional regulator, we inferred CDX2 regulatory activity using the Virtual Inference of Protein-activity by Enriched Regulon analysis (VIPER) algorithm (*27*), based on a CRC regulon network. CDX2 activity was strongly positively correlated with upstream SLC39-mediated zinc influx and downstream CD24 expression in both the TCGA and CPTAC cohorts (Fig. 2C). Next, we investigated the epigenomic architecture of the *CD24* locus to determine the mechanistic basis of its transcriptional activation (Fig. 2D). In the Caco-2 CRC cell line, which exhibits a dominant CMS2 signature while retaining partial CMS4-associated features in transcriptome-based CMS analyses (*28*), chromosome conformation capture (Hi-C) chromatin interaction data (*29*) demonstrated that the *CD24* promoter and a previously annotated distal enhancer (*30*) reside within the same topologically associated domain (TAD) (Fig. 2D, top). This enhancer region displayed enrichment of active chromatin markers (*31*), including H3K4me1, H3K36me3, and H3K27ac, in Caco-2 cells (Fig. 2D, middle). A single-cell assay based on transposase-accessible chromatin sequencing (scATAC-seq) data from 18,275 cells from three patients with CRC (*32*) further revealed high chromatin accessibility at this enhancer region, specifically in cancer cells, compared to that in other cell types (Fig. 2D, bottom). To assess enhancer activity in patient samples, we analyzed transposase-accessible chromatin sequencing (ATAC-seq) data from 25 patients with TCGA-COAD (*33*) and observed significant positive correlations between CD24 enhancer accessibility and upstream regulatory signals, including SLC39-mediated zinc influx, CDX2 activity, and CD24 expression (Fig. 2E). Furthermore, motif-enrichment analysis confirmed the significant binding of CDX2 to the putative enhancer region, supporting its direct role in CD24 activation (Fig. 2F). Considering prior evidence that zinc-dependent phosphorylation modulates CDX2 transcriptional output, we examined phosphoproteomic datasets (*34*) to evaluate CDX2 posttranslational regulation. Although CDX2 expression and activity were enriched in CRC, they were not restricted to CRC (fig. S2, F to H), prompting the phosphosite-specific evaluation of CRC. Among the detectable CDX2 phosphosites, S176 showed the strongest association with zinc influx, CDX2 activity, and CD24 expression (Fig. 2G and fig. S2I). Candidate kinases, including MEK2, PKCε, and DNA-PKcs, were enriched in zinc-associated networks and preferentially correlated with the CDX2 S176 region (fig. S2, J and K), which is consistent with prior links between zinc and these kinase pathways (*35*–*37*). Last, AlphaFold3-based structural modeling and HDOCK protein-DNA docking supported high-confidence CDX2 binding to the CD24 enhancer (*38*, *39*). Notably, S176 phosphorylation increased the binding affinity of the predicted CDX2 enhancer (Fig. 2, H and I). Together, these analyses define a zinc-responsive enhancer circuit in which SLC39-driven zinc influx amplifies CDX2 activity and induces CD24 expression, thereby establishing a metabolic route for immune-evasive programming in CRC.

**Fig. 2.**
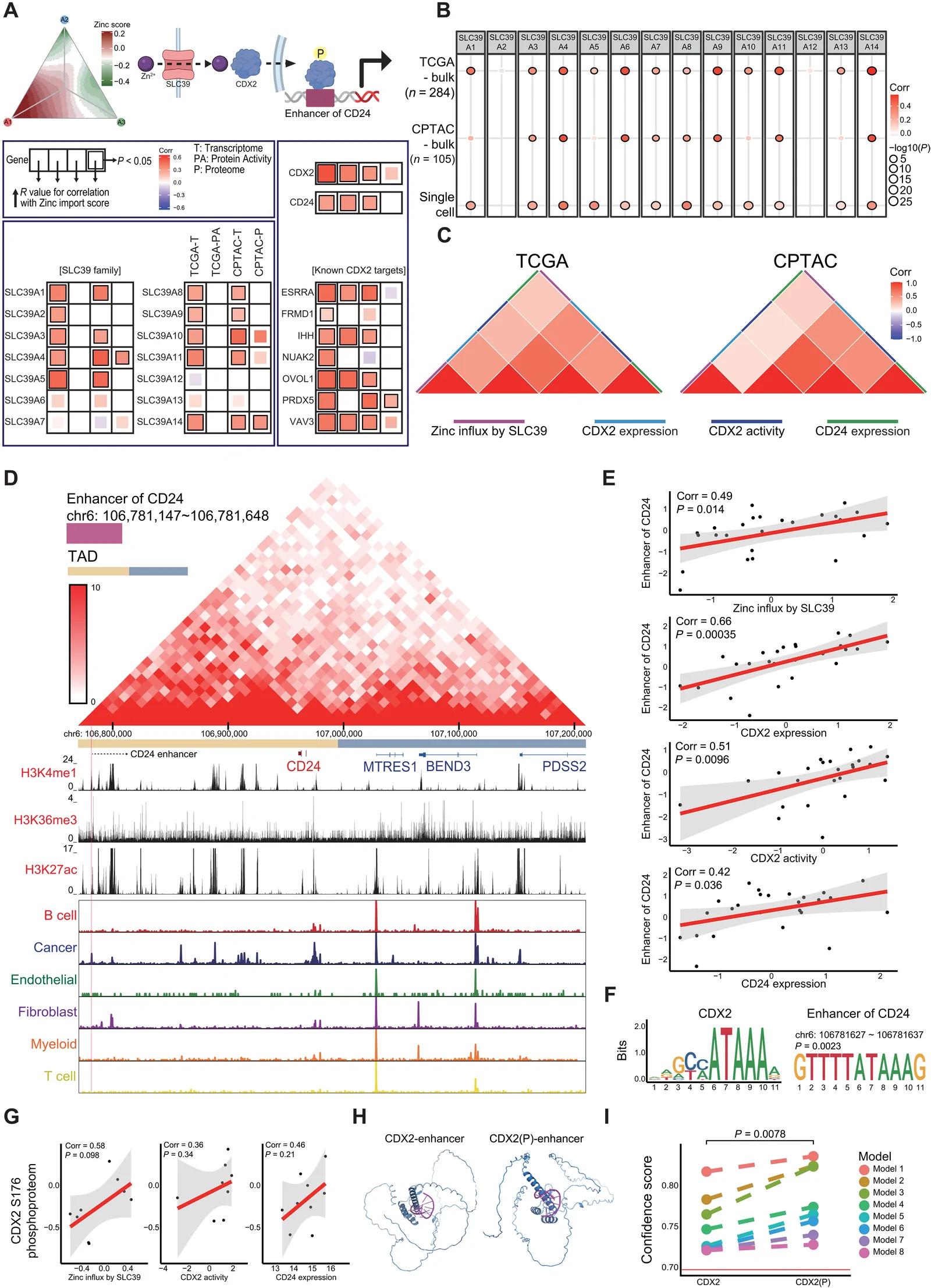
Identification of the zinc-SLC39-CDX2 axis and CD24 enhancer regulated by CDX2 activity. (**A**) Integrative correlation analysis of the SLC39-CDX2-CD24 axis using TCGA transcriptomic (*n* = 288) and CPTAC proteomic data (*n* = 90). (**B**) Correlation between CNV and gene expression for SLC39 family members using bulk-level TCGA/CPTAC data and single-cell inferred CNV data by inferCNV from CRC samples. (**C**) Zinc influx by SLC39 score was calculated as the mean normalized expression of nine membrane-localized SLC39 genes (*SLC39A1*, *A2*, *A3*, *A4*, *A5*, *A6*, *A8*, *A10*, and *A14*). Pairwise correlations among zinc influx, CDX2 expression/activity, and CD24 expression were assessed in TCGA and CPTAC cohorts. (**D**) Epigenomic profiling of a putative CD24 enhancer. Caco-2 Hi-C showed enhancer-CD24 localization within the same TAD. Caco-2 ChIP-seq showed H3K4me1, H3K36me3, and H3K27ac signals, and CRC scATAC-seq (*n* = 3) showed accessibility across annotated cell types. (**E**) Correlation between chromatin accessibility of the CD24 enhancer (TCGA-ATAC dataset) and zinc influx, CDX2 expression and activity, and CD24 expression, highlighting a coordinated regulatory relationship. (**F**) Motif analysis within the CD24 enhancer region identified canonical binding sequences for CDX2. (**G**) Correlation analysis across patients from the CPTAC CRC cohort (*n* = 9) revealed that phosphorylation levels of CDX2 at serine-176 were positively associated with zinc influx by SLC39 scores (*P* = 0.098), CDX2 activity scores (*P* = 0.34), and CD24 expression scores (*P* = 0.21). (**H**) Structural modeling of CDX2 and phosphorylated CDX2 at serine-176 (CDX2-pS176) using AlphaFold3, illustrating DNA-binding configurations. (**I**) Protein-DNA docking analysis using HDOCK for CDX2 and CDX2-pS176 against the CD24 enhancer sequence. Confidence scores were calculated from docking scores using the HDOCK empirical function. Top 8 models were evaluated, and CDX2-pS176 showed higher confidence scores than CDX2 (*P* = 0.0078). Created in BioRender. Lab, S. (2026) https://BioRender.com/6c6ih16.

### Cancer cell–restricted and context-dependent CD24 induction via SLC39-mediated zinc influx

Most of the signaling cascades identified in this study were derived from bulk tissue data, which consist of heterogeneous cell populations. To resolve cell type–specific signaling, we analyzed the impact of zinc influx mediated by SLC39 overexpression at the single-cell level using a transcriptomic dataset comprising 174,547 single cells from 26 patients with CRC (*25*). Canonical cell types were annotated based on established marker genes (Fig. 3A and fig. S3A), and the expression patterns of zinc influx–mediated SLC39 signaling, leading to the up-regulation of CD24 expression, were examined across cell types (Fig. 3B, top). Correlation analyses among the signaling factors were performed in a cell type–specific manner (Fig. 3B, bottom). These analyses revealed a strong and selective association between SLC39-driven zinc influx and CDX2 activity and CD24 expression in malignant epithelial cells, whereas nonmalignant compartments exhibited minimal or marginal correlations, consistent with the limited resolution of the scRNA-seq data. To address this limitation, we conducted a metacell analysis using SEACells (*40*), which mitigates scRNA-seq data sparsity while preserving cellular heterogeneity by defining compact and well-separated metacells (Fig. 3C and fig. S3B). This approach enabled the detection of robust CD24 overexpression driven by zinc influx via SLC39 exclusively in cancer cells (Fig. 3D), accompanied by the most significant positive correlation in cancer cells compared to that in other cell types (Fig. 3E). To validate these observations functionally, we overexpressed SLC39A4 or SLC39A5 (fig. S3C), the two SLC39 family members most strongly associated with zinc influx in our multi-omics analyses, (Fig. 2A) in the CRC cell line LS174T. LS174T represents a non-CMS2 subtype (*41*) and thus provides a distinct cellular context to test whether the forced activation of SLC39 signaling is sufficient to induce downstream zinc influx and CD24 expression. The overexpression of either transporter significantly increased intracellular zinc levels, as measured based on FluoZin-3 fluorescence (Fig. 3F). Concomitantly, the overexpression of either gene up-regulated CD24 expression at the transcript and protein levels (Fig. 3, G and H) and increased membrane-localized CD24, as confirmed via flow cytometry (Fig. 3I and fig. S3D). These cells displayed enhanced CDX2 activity and nuclear localization despite unchanged total CDX2 protein levels (Fig. 3G and fig. S3, E to G). In addition, CDX2 overexpression increased membrane-localized CD24 expression, supporting the direct role of CDX2 in promoting CD24 expression (Fig. 3J). To assess context dependency, we performed analogous experiments using the non–CMS2-like CRC cell line HCT116 (*41*). Despite achieving comparable SLC39A5 overexpression, the induction of CD24 was substantially attenuated relative to that in LS174T cells, potentially reflecting lower basal CDX2 expression in HCT116 cells (fig. S3H). To further validate the SLC39-CDX2-CD24 axis in a CMS2-like CRC model, we examined NCIH508 cells, which exhibit higher expression of SLC39A5, CDX2, and CD24 than HCT116 and LS174T cells (fig. S3, I to K). In this context, both SLC39A5 knockdown and CDX2 knockout reduced CD24 expression (Fig. 3, K to N). Thus, the SLC39-CDX2-CD24 axis appears to operate as a context-dependent, metabolically amplified program, rather than as a universal mechanism across all CRC cells. CD24 has been established as a potent “don’t eat me” signal analogous to CD47 and is broadly overexpressed across multiple tumor types (*23*). However, a comparative analysis demonstrated that CD24, but not CD47, was selectively enriched in cancer cells and exhibited a significant positive correlation with SLC39-mediated zinc influx in LS174T CRC cells (fig. S3, L to N). Consistently, the overexpression of SLC39A4 or SLC39A5 induced CD24 but not CD47 transcription in CRC cells (Fig. 3O). Together, these findings show that SLC39-mediated zinc influx selectively induces CD24 expression in malignant cells, defining a CD47-independent immune-evasion program.

**Fig. 3.**
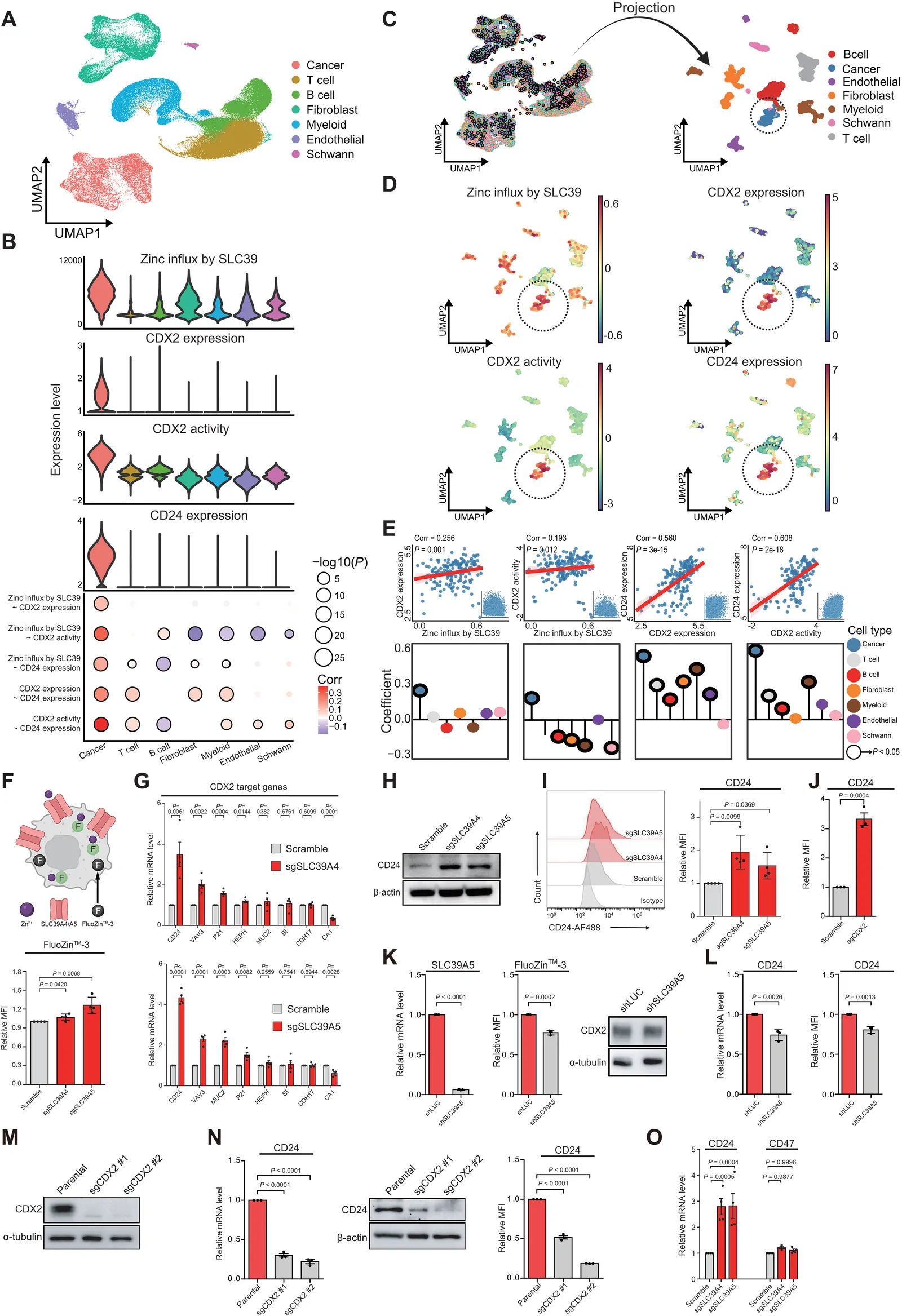
Single-cell analyses identify cancer cell–specific activation of the SLC39-CDX2-CD24 axis with functional validation. (**A**) UMAP of 174,547 CRC cells by major cell type. (**B**) Cell type–specific SLC39 zinc influx, CDX2 expression/activity, CD24 expression, and correlations. (**C** and **D**) Metacell UMAP and feature distributions. (**E**) Cancer metacell correlations with single-cell insets. (**F**) Quantification of intracellular zinc levels through FluoZin-3 fluorescence in SLC39A4- and SLC39A5-overexpressing cells. Fluorescence was quantified as MFI (mean fluorescence intensity) per cell (*n* = 4). (**G**) Quantitative RT-PCR analysis of CDX2 target genes in SLC39A4- and SLC39A5-overexpressing cells (*n* = 4). (**H** and **I**) Western blot analysis of CD24 protein (H) and flow cytometric analysis of cell-surface CD24 (I) (*n* = 4). (**J**) Flow cytometry analysis of CD24 in CDX2-overexpressing LS174T cells (*n* = 3). (**K**) Quantitative RT-PCR analysis of SLC39A5 expression (left), quantification of intracellular zinc through FluoZin-3 (center), and Western blot of CDX2 protein levels in doxycycline-inducible SLC39A5-knockdown NCIH508 cells (right) (*n* = 3). shRNAs were induced with the addition of doxycycline (250 ng/ml) for 4 days. (**L**) Quantitative RT-PCR (left) and flow cytometry (right) analyses of CD24 expression in SLC39A5-knockdown NCIH508 cells (*n* = 3). (**M**) Validation of CDX2 knockout in NCIH508 cells. (**N**) Quantitative RT-PCR (left), Western blot (center), and flow cytometry (right) analyses of CD24 expression at the transcript, total protein, and membrane localization levels, respectively, in CDX2-knockout cells (*n* = 3). (**O**) Quantitative RT-PCR analysis of CD24 and CD47 in SLC39A4- and SLC39A5-overexpressing cells (*n* = 4). In (F), (G), (I), (J), (K), (L), (N), and (O), data are presented as mean ± SEM. Unpaired two-tailed *t* test was used for (F), (G), (I), (J), (K), (L), and (N). Two-way ANOVA followed by Tukey’s multiple comparisons test was used for (O). Created in BioRender. Lab, S. (2026) https://BioRender.com/8kt9m8t.

### SLC39-mediated zinc influx directs the cell state trajectory toward CMS2-like cancer cells associated with poor survival and immunotherapy resistance

To investigate the influence of SLC39-mediated zinc influx on cancer cell state dynamics, we applied the Palantir algorithm (*42*) to the single-cell transcriptomic data (fig. S4A). CMS-based subtyping was performed to annotate the inferred cell state trajectories (Fig. 4A, left). Three terminal states were identified based on pseudotime and entropy (Fig. 4A, right), with terminal state 1 converging into a subset of CMS2-like cancer cells and terminal states 2 and 3 remaining heterogeneous or unclassified (fig. S4B). An analysis of the SLC39-driven zinc influx components revealed selective overexpression or TF activation in terminal state 1 (Fig. 4B). Gene trend analysis further confirmed that signaling cascades downstream of SLC39-mediated zinc influx were specifically up-regulated along the trajectory toward terminal state 1 (Fig. 4C). Notably, *CD24* expression exhibited a delayed onset relative to the upstream regulators, consistent with its function as a downstream effector of zinc-induced *CDX2* activation. Pathway enrichment analysis of terminal state-specific expression signatures confirmed that terminal state 1 significantly activated or repressed CMS2 subtype–associated pathways (Fig. 4D). Given the immunosuppressive nature of the CMS2-like tumors identified in our archetype analysis (Fig. 1D), we examined the immunomodulatory potential of cancer cells in terminal state 1. Notably, CD24 emerged as the top-ranked immunomodulator overexpressed in this state, reinforcing its role as a key effector of the SLC39-driven zinc influx axis (Fig. 4E and fig. S4C). Expression of the canonical “don’t eat me” signal CD47 was not significantly up-regulated in terminal state 1.

**Fig. 4.**
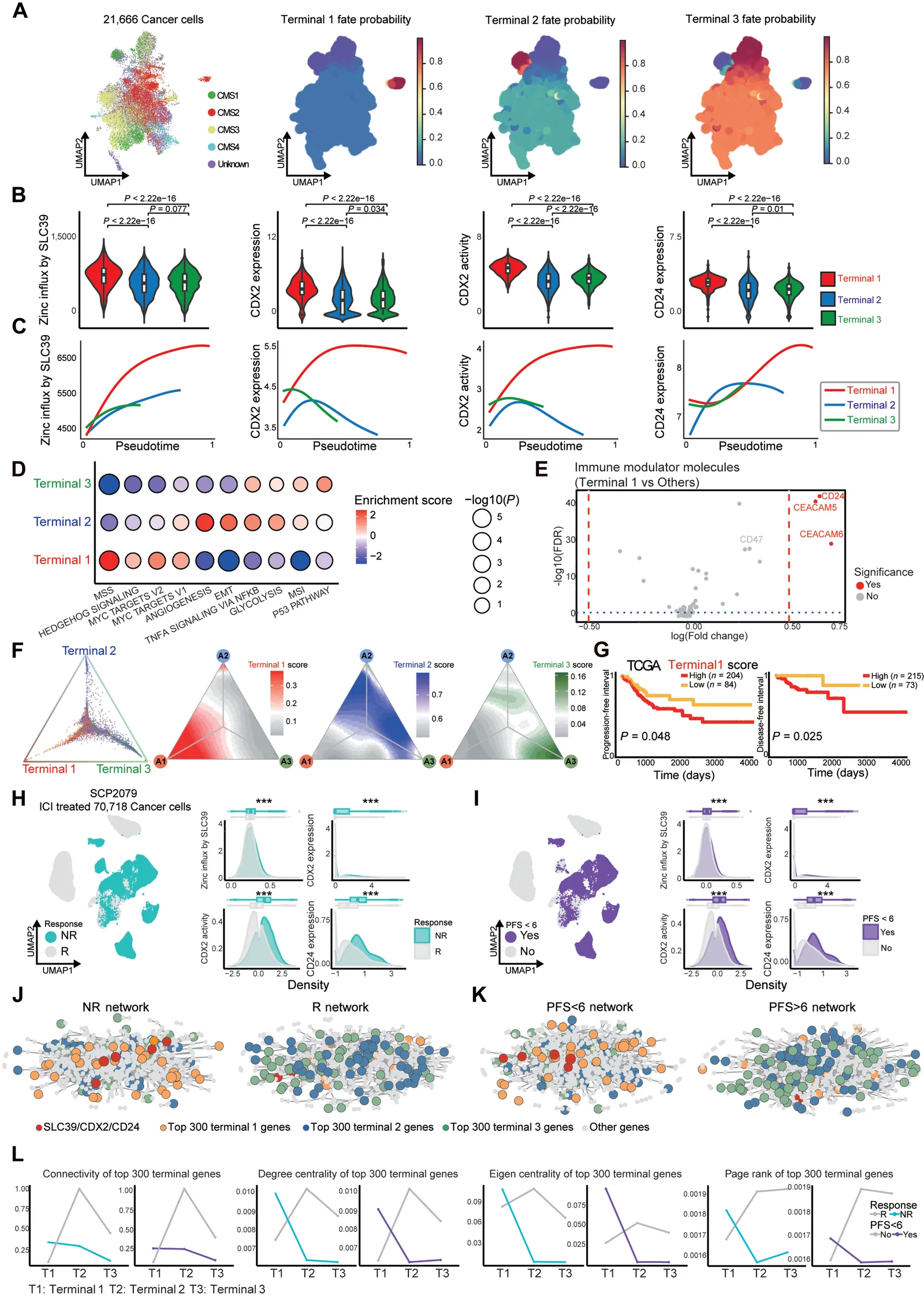
Trajectory-informed characterization of zinc signaling–driven terminal states reveals a CD24^+^ immune-evasive fate associated with poor prognosis and immunotherapy resistance. (**A**) UMAP visualization of 21,066 cancer cells. In the left panel, cells are colored by CMS classification (CMS1 to CMS4). In the right three panels, cells are colored according to inferred fate probabilities toward each of the three terminal states, as estimated by Palantir, with color intensity indicating a continuous probability scale. (**B**) Terminal fate-wise expression of zinc influx by SLC39, CDX2 expression/activity, and CD24 expression. (**C**) Smoothed gene expression trends along the trajectory toward each terminal state. (**D**) Terminal-specific enrichment analysis of hallmark and CRC-relevant pathways. (**E**) Terminal 1 versus other terminal states differential expression analysis of curated immune modulatory genes, using log_2_ fold change >0.5 as the cutoff. (**F**) Projection of trajectory-inferred terminal state programs onto TCGA-COAD bulk transcriptomes. Marker gene signatures from terminal 1 to 3 cancer cell populations identified by single-cell RNA-seq trajectory analysis were applied to TCGA bulk RNA-seq data using gene signature–based deconvolution. The left panel shows the distribution of terminal 1 to 3 programs across CMS subtypes, whereas the right panel visualizes enrichment of each terminal program across archetypes A1 to A3 defined by ParetoTI-based archetype inference from TCGA multi-omics integration. (**G**) Kaplan-Meier analysis of PFI and DFI in TCGA-COAD stratified by terminal 1 scores. (**H** and **I**) SCP2079 CRC single-cell UMAP and density plots from pre-ICI samples, annotated by response status (NR versus R) or PFS group (PFS < 6 months versus ≥ 6 months); feature distributions were compared using two-sided *t* tests (*P* < 2.2 × 10^−12^). (**J** and **K**) Condition-specific gene networks were constructed using scHumanNet for NR versus R samples and for PFS < 6 months versus PFS ≥ 6 months groups. (**L**) Network metrics for terminal 1 to 3 markers.

An extension of single-cell–derived findings to patient cohorts was achieved by constructing a terminal state signature matrix using the CellRank algorithm (*43*), which enabled the calculation of terminal state probability scores from bulk transcriptomic datasets (Fig. 4F, left). To clarify the relationship between bulk-derived archetypes and single-cell–derived terminal states, we projected cancer cell–derived terminal state signatures onto 258 TCGA-COAD patient samples. This analysis revealed that the terminal state 1 probability scores were significantly enriched in archetype 1 patients, characterized by CMS2-like features and activated SLC39-mediated zinc influx signaling (Fig. 4F, right). Thus, terminal state 1 represents the cancer cell–intrinsic counterpart of the bulk-defined archetype 1 state, rather than an independent classification system. In contrast, terminal states 2 and 3 were heterogeneously distributed across archetypes 2 and 3. Survival analysis demonstrated that patients with higher terminal state 1 probability scores had significantly shorter progression-free and disease-free intervals (Fig. 4G). This association was specific to terminal state 1, as no significant survival differences were observed for terminal state 2 or 3 (fig. S4D). To investigate the relevance of this state to the immunotherapy response, we analyzed single-cell transcriptomes from 70,718 cancer cells derived from patients with CRC treated with programmed cell death protein 1 (PD-1) inhibitors (*44*) (Fig. 4, H and I). Intriguingly, the overexpression or activation of components of the SLC39-driven zinc influx pathway was consistently associated with nonresponse to therapy and shorter progression-free survival (PFS). To elucidate the regulatory landscape underlying these clinical associations, we constructed patient-specific cancer cell networks using scHumanNet (*45*) stratified by the clinical response or PFS status. The mapping of terminal state marker genes and core components of the SLC39-driven zinc influx pathway onto these networks revealed the significant enrichment of terminal state 1 markers and zinc influx components within the nonresponder and short-term survivor networks (Fig. 4, J and K). Conversely, terminal state 2 and 3 markers were predominantly enriched in the responder and long-term survivor networks. This distribution pattern remained consistent, regardless of the number of marker genes included in the analysis (fig. S4E). A systematic importance evaluation using centrality metrics further demonstrated that terminal state 1 markers and SLC39-driven zinc influx components exhibited higher importance scores than other marker genes, specifically in the nonresponder and short-survivor networks (Fig. 4L). This pattern was robust to variations in the marker gene-selection criteria (fig. S4F). Collectively, these findings demonstrate that the SLC39-driven zinc influx axis drives a CMS2-like cancer cell state marked by CD24 overexpression, which is clinically associated with poor prognosis and resistance to immunotherapy.

### CD24 mediates pleiotropic immune evasion signaling in the TME through SIGLEC10 interactions

Because archetype 1 tumors show CMS2-like immune-desert features, we next investigated how CD24-overexpressing tumor cells communicate with immune populations in the TME. Ligand-receptor analysis, with cancer cells as the sender population, identified CD24-SIGLEC10 as a tumor-selective interaction with myeloid cells (Fig. 5A and fig. S5A). Among these, CD24 emerged as a tumor-selective ligand, predicted to interact exclusively with SIGLEC10 receptors expressed on myeloid cells, suggesting a specialized axis of immune suppression mediated by CD24-SIGLEC10 engagement. Chromatin accessibility analysis further revealed that the *SIGLEC10* promoter was epigenetically active exclusively in myeloid populations from samples from patients with CRC (*32*) (fig. S5B). To identify specific myeloid subpopulations expressing SIGLEC10, we performed subclustering using lineage-specific markers (fig. S5C). This analysis revealed the significant overexpression of SIGLEC10 in monocytes, resident tissue macrophage-like cells (RTMCs), monocyte-derived macrophages (moMCs), and monocyte-derived dendritic cells (moDCs) compared to levels in other myeloid subsets (Fig. 5B). Myeloid-lineage cells originate from distinct progenitors: RTMCs are derived from erythromyeloid progenitors, whereas monocytes, moMCs, and moDCs are derived from hematopoietic stem cells (Fig. 5C). Cell-state trajectory analysis using the Palantir algorithm (*42*) revealed bifurcating developmental paths from monocytes toward moMC and moDC lineages (Fig. 5D and fig. S5D). Gene trend profiling along these trajectories showed the progressive up-regulation of SIGLEC10 expression, confirming lineage development through stage-specific marker expression (Fig. 5E). We next inferred downstream programs associated with CD24-SIGLEC10 signaling in SIGLEC10^+^ myeloid subsets. In moMCs, CD24 exhibited high ligand activity and selectively engaged SIGLEC10 (Fig. 5F, left), leading to the activation of downstream transcriptional programs exclusively expressed in moMCs (Fig. 5F, right). Functional enrichment analysis of these target genes revealed strong associations with the negative regulation of macrophage activation, phagocytosis, endocytosis, and immune responses, which are hallmarks of the “don’t eat me” signal (Fig. 5G). Similarly, moDCs exhibited a parallel immune-repressive signaling trend, although the specific downstream gene sets differed from those in moMCs (fig. S5, E and F). Beyond moMCs and moDCs, RTMCs also interacted with cancer cells via CD24-SIGLEC10, but their downstream signaling profiles diverged, predominantly influencing cell adhesion and chemotaxis pathways (Fig. 5, H and I). These results suggest that tumor-derived CD24 engages SIGLEC10^+^ moMCs, moDCs, and RTMCs to induce pleiotropic programs involving phagocytosis suppression and adhesion remodeling.

**Fig. 5.**
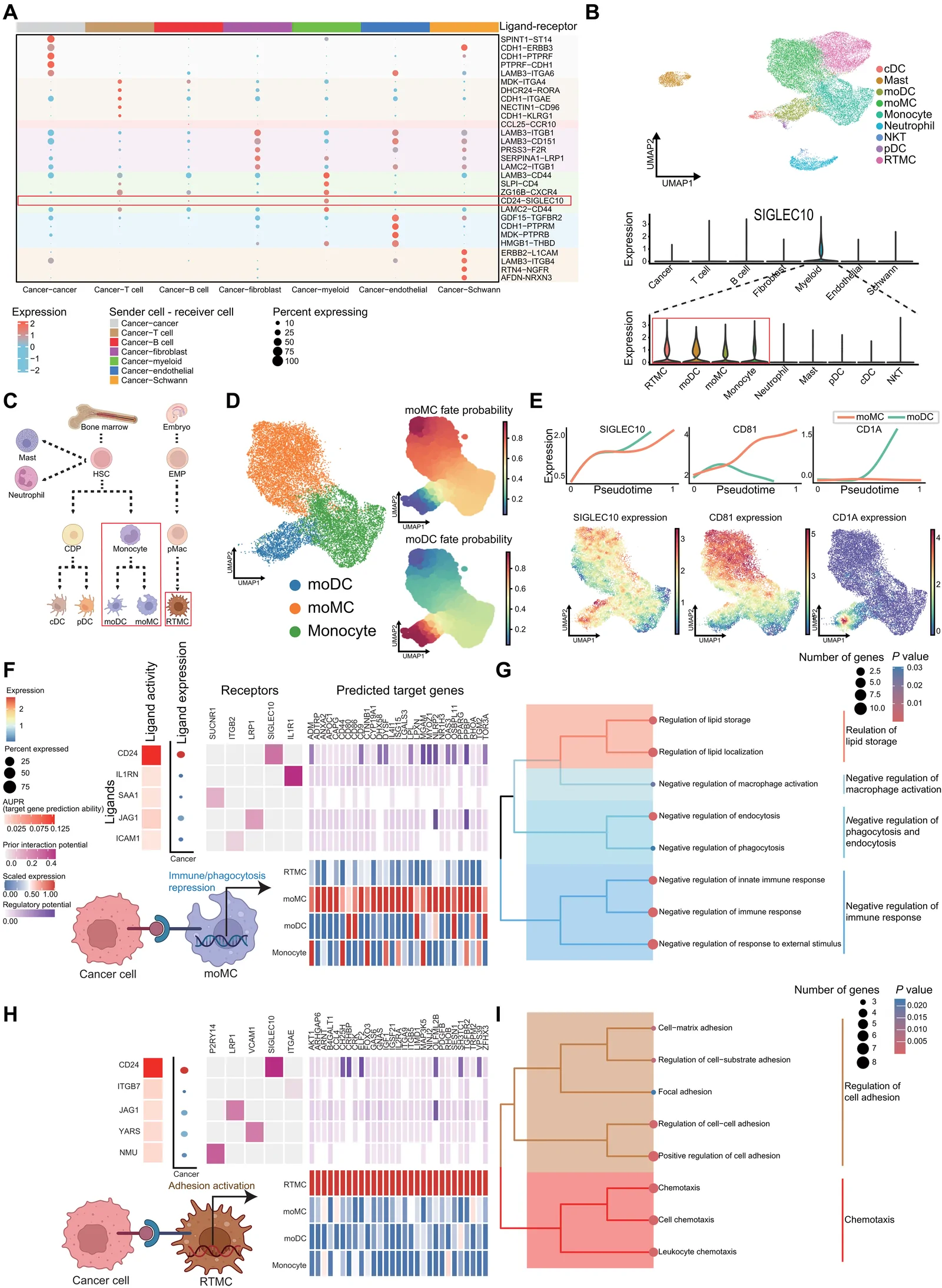
Pleiotropic CD24-SIGLEC10 signaling bifurcates into immunosuppressive and adhesive programs in distinct macrophage lineages. (**A**) Cell-cell interaction profiling using NICHES across CRC single-cell transcriptomes was performed by setting cancer cells as the sender and evaluating interactions with all other major cell types. For each cancer-to-receiver pair, top-ranking ligand-receptor interactions were identified, and the top five interactions were selected based on specificity and expression strength. (**B**) UMAP-based classification of myeloid cells into fine-grained subtypes, including monocyte-derived macrophages (moMC), tissue-resident macrophages (RTMC), monocytes, and dendritic cells (cDC, pDC, and moDC), reveals distinct lineage states. (**C**) Schematic of myeloid developmental trajectories illustrating that both moMC and RTMC arise from distinct ontogenies: moMC from circulating monocytes, and RTMC from embryonic precursors, establishing the basis for functional divergence. Cell populations known to exhibit high SIGLEC10 expression [as shown in (B)] are highlighted with red boxes. (**D**) Diffusion-based pseudotime and fate probability analysis of monocytes, moMC, and moDC revealed bifurcating differentiation paths, each with distinct lineage commitment. (**E**) Gene expression trends along both moMC and moDC pseudotime trajectories demonstrated progressive up-regulation of SIGLEC10, indicating that SIGLEC10 activation occurs along both monocyte-derived macrophage and dendritic lineages. Lineage-specific markers *CD81* (moMC) and *CD1A* (moDC) were shown as references. (**F** and **G**) NicheNet analysis of cancer-moMC pairs identified the top five ligands ranked by ligand activity AUPR. CD24 was included among the top-ranked ligands, and predicted target genes were used for expression visualization and functional enrichment analysis. (**H** and **I**) The same NicheNet workflow was applied to cancer-RTMC pairs, followed by predicted target gene expression and pathway enrichment analyses. Created in BioRender. Lab, S. (2026) https://BioRender.com/qcu9zcs.

### CD24-SIGLEC10–enriched niches define immunorepressive microenvironments in CRC

To evaluate whether the pleiotropic cellular interactions delineated in our analysis are spatially organized within tumor tissues, we analyzed the spatial transcriptomes of four samples from patients with CRC (*46*). Cell-type deconvolution was performed using SPOTlight (*47*) with canonical marker genes (Fig. 6A). Spatial domains enriched in CD24-SIGLEC10 interactions were identified using SPATA2 (*48*), which enabled the delineation of pleiotropic signaling regions (Fig. 6B). Notably, cancer cells, moMCs, moDCs, and RTMCs, which are key cell types engaged in CD24-SIGLEC10 signaling, were consistently enriched at the tumor periphery near these interaction hotspots (Fig. 6, A and B, bottom). To investigate the functional relevance of this cellular ecosystem, we quantified the activity of core components of the SLC39-mediated zinc influx axis relative to their spatial proximity to CD24-SIGLEC10–enriched areas. All signaling components demonstrated peak activity adjacent to these regions, with progressive attenuation observed with increasing distances (fig. S6, A and B). Consistent spatial gradients were also observed for immune evasion and cell adhesion pathways, with maximal activation near the interaction zones and a gradual decline further away (Fig. 6, C and D), providing functional validation of these spatially defined immunoregulatory microenvironments. On the basis of these observations, we characterized a cellular niche representing an immunosuppressive microenvironmental ecosystem mediated by CD24-SIGLEC10 pleiotropic interactions, encompassing the signaling cascades of CD24 overexpression in cancer cells (Fig. 6E, bottom, red background box), CD24-SIGLEC10 interactions (Fig. 6E, bottom, blue background box), and downstream pleiotropic signals regulating immune evasion and cell adhesion (Fig. 6E, bottom, green background box). Using the FindClusters function of Seurat (*49*), we identified 10 to 14 distinct cellular niches across the four CRC tissue samples (Fig. 6E and fig. S6C). Two of the four tissue samples exhibited the immunosuppressive cellular niches that we characterized, with concordant activation patterns of signaling cascades mediated by CD24-SIGLEC10 interactions in the peripheral tissue areas (Fig. 6E, top). In contrast to these niches, the CD47-SIRPα interaction—a putative major “don’t eat me” signal—showed minimal or only partial activation in these regions (fig. S6D). Collectively, these spatial transcriptomic analyses identified CD24-SIGLEC10–enriched immunosuppressive niches in a subset of CRC tissues and highlighted patient-level heterogeneity in their spatial organization and prevalence.

**Fig. 6.**
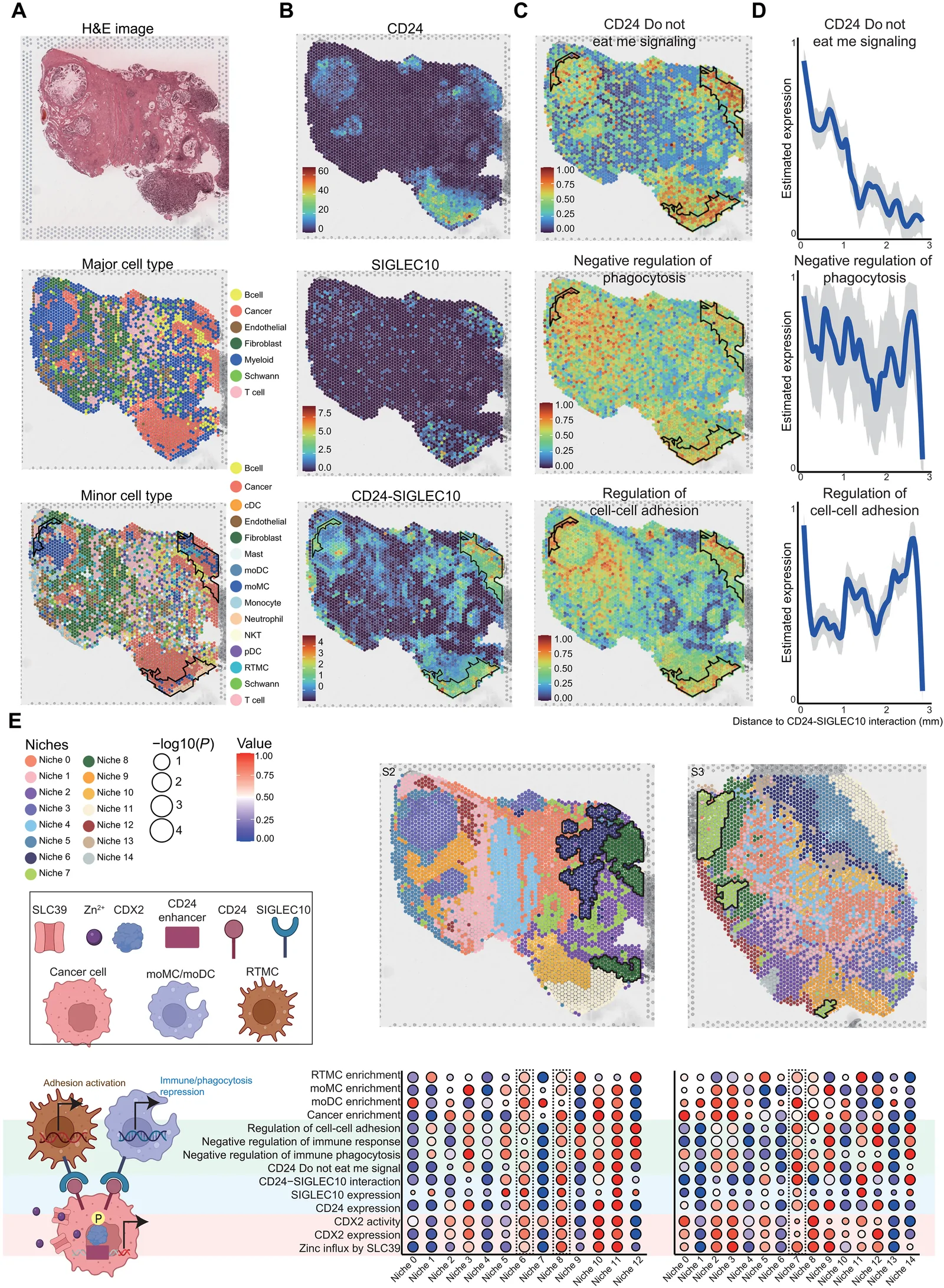
Spatial mapping of CD24-SIGLEC10 interaction and associated signaling pathways in CRC tissue sections. (**A**) Representative CRC Visium spatial transcriptomics section (one of four patients) with matched H&E image and SPOTlight-based major/minor cell-type deconvolution using Fig. 3A scRNA-seq annotations as reference. CD24-SIGLEC10 interaction–enriched regions identified by NICHES and SPATA2 are outlined with black contours. The tissue image used in (A) was obtained from the Gene Expression Omnibus (GEO) database under accession GSE225857, specifically sample GSM7058757 (*46*). (**B**) Spatial distribution of CD24 and SIGLEC10 expression levels across spatial spots, quantified from normalized expression matrices. (**C**) Spatial projection of inferred pathway activities based on known gene sets associated with CD24-mediated “Don’t eat me” signaling, negative regulation of phagocytosis, and regulation of cell-cell adhesion. (**D**) Enrichment scores for the gene sets shown in (C) were plotted relative to their distance from spatial annotations enriched for CD24-SIGLEC10 interaction. Line plots represent smoothed expression trends estimated by local polynomial regression (loess), with distance calculated from the annotated border of the interaction-enriched region. (**E**) Identification of spatial niches based on unsupervised clustering of spatial transcriptomes. The left panel shows schematic representations of the molecular features mapped in the dataset, including zinc influx by SLC39, CDX2 expression, CDX2 activity, CD24 and SIGLEC10 expression, CD24-SIGLEC10 interaction scores, downstream pathway signatures, and enrichment scores of relevant cell types. The right panel shows spatial domains annotated as distinct niches across two representative samples (S2 and S3), where each domain was scored for enrichment of the aforementioned molecular features. Niches enclosed by black dotted boxes represent regions where all axes of the zinc-CDX2-CD24-SIGLEC10 signaling cascade are concurrently enriched. Created in BioRender. Lab, S. (2026) https://BioRender.com/c3djcpu.

### Conserved pleiotropic CD24-SIGLEC10 signaling defines immune-evasive myeloid networks associated with immunotherapy resistance

We investigated whether pleiotropic immune-evasive signaling mediated by CD24-SIGLEC10 interactions is preserved at the patient level. To this end, we derived cell type–specific gene signatures from the top 50 moMC, moDC, and RTMC markers identified in single-cell transcriptomic datasets (*50*). These gene sets were mutually exclusive and independent of downstream CD24-SIGLEC10 signaling modules (fig. S7A). Using these signatures, we computed the enrichment scores for moMCs and RTMCs across TCGA-COAD samples and stratified the patients into subgroups based on the combinatorial high (+) or low (–) enrichment status (Fig. 7A). The potential for intercellular CD24-SIGLEC10 interactions was assessed using the LIANA module from the DecoupleR framework (*51*), which quantifies ligand-receptor coexpression relative to background distributions. Patients exhibiting concurrent high enrichment of both moMCs and RTMCs demonstrated the strongest predicted CD24-SIGLEC10 interaction potential (Fig. 7B). To assess the functional consequences of these interactions, we analyzed gene modules associated with CD24-SIGLEC10 activity, encompassing immune-evasive “don’t eat me” signals and adhesion-regulatory pathways. These modules showed robust positive correlations with moMC and RTMC enrichment scores despite minimal gene overlap with the respective marker sets (Fig. 7C and fig. S7A). This association was consistent when moDCs were substituted with moMCs (fig. S7, B to D). In addition, the immunosuppressive cellular landscape identified in patients in TCGA was independently recapitulated in the CPTAC CRC cohort (fig. S7E), highlighting the robustness and translational significance of these findings. To mechanistically recapitulate these observations, we used THP-1 cells differentiated into moMC-like or RTMC-like lineages using distinct polarization protocols (Fig. 7D). Each protocol selectively enhanced the marker expression of the corresponding myeloid subtype. To test whether CD24-SIGLEC10 engagement suppresses phagocytic activity, LS174T CRC cells were cocultured with differentiated moMC-like cells and phagocytosis was quantified (Fig. 7E). SLC39A4- or SLC39A5-overexpressing CRC cells exhibited significantly reduced phagocytosis compared with control cells, and this effect was reversed by anti-CD24 antibody treatment, confirming that suppression occurred through the CD24-SIGLEC10 interaction. Similarly, CDX2-overexpressing CRC cells showed reduced phagocytosis, further supporting the functional contribution of the SLC39-CDX2-CD24 axis to CD24-dependent antiphagocytic signaling in this context (Fig. 7F). Motivated by these mechanistic insights, we hypothesized that the expression of key components within the CD24-SIGLEC10 axis could stratify immunotherapy responses. To test this, we integrated five independent immune checkpoint inhibitor (ICI)–treated CRC cohorts (*n* = 166) encompassing cases of both MSS and microsatellite instability (MSI) and applied batch correction to harmonize the expression profiles (Fig. 7G and fig. S7F). A Gradient Boosting Classifier was trained on a biologically informed seven-gene signature consisting of *SLC39A2*, *SLC39A4*, *SLC39A5*, *SLC39A10*, *CDX2*, *CD24*, and *SIGLEC10*, which were selected for their central role in zinc-driven immunosuppression. This model achieved the highest predictive accuracy among all benchmarked classifiers, with an area under the curve (AUC) of 0.728 in an independent testing set, outperforming established biomarkers of adaptive immunity, stromal resistance, and tumor-intrinsic evasion (*52*–*59*) (Fig. 7H). Together, these findings define a conserved pleiotropic immunosuppressive circuit orchestrated by SLC39-driven zinc influx and CD24-SIGLEC10 engagement. This pathway shapes the immune-evasive TME, suppresses macrophage phagocytosis, and serves as a clinically actionable predictor of ICI responses in CRC.

**Fig. 7.**
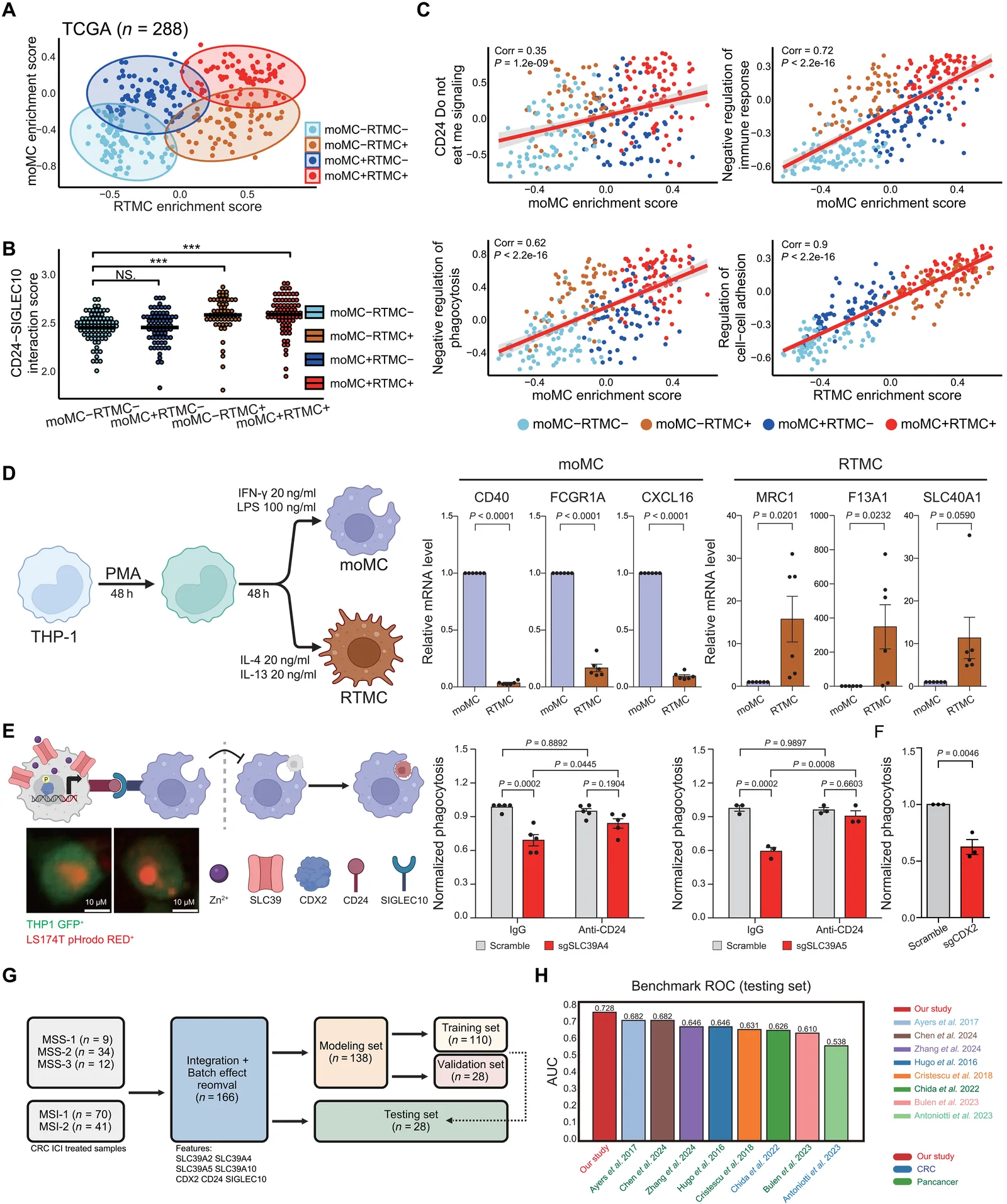
Pleiotropic CD24-SIGLEC10 signaling promotes divergent macrophage programs and correlates with poor immune outcome, functionally validated through targeted assays. (**A**) TCGA-COAD samples (*n* = 288) were scored using moMC and RTMC markers and clustered into four subtypes. (**B**) CD24-SIGLEC10 scores were inferred using DecoupleR/LIANA and compared across subtypes. (**C**) Correlations between moMC/RTMC scores and their associated functional pathways. (**D**) Schematic diagram of lineage-selective differentiation of THP-1 cells into moMC-like or RTMC-like macrophages and quantitative RT-PCR analysis of representative moMC and RTMC markers (*n* = 6). (**E**) Schematic diagram of phagocytosis assay using moMC-like differentiated THP-1 cells expressing GFP and pHrodo Red–treated LS174T CRC cells (top left) and cellular images of GFP^+^ THP-1 cells that have phagocytosed pHrodo Red–treated LS174T cells (bottom left). Graph shows quantification of the ratio of pHrodo Red–positive GFP^+^ THP-1 cells cocultured with SLC39A4- or SLC39A5-overexpressing LS174T cells in the presence of either IgG or anti-CD24 antibodies (right) (*n* = 5 and *n* = 3). (**F**) Phagocytosis assay using moMC-like differentiated THP-1 cells expressing GFP and pHrodo Red–treated CDX2-overexpressing LS174T cells (*n* = 3). (**G**) Five ICI-treated CRC cohorts—MSS1 (*n* = 9), MSS2 (*n* = 34), MSS3 (*n* = 12), MSI1 (*n* = 70), and MSI2 (*n* = 41)—were integrated and batch corrected (*n* = 166). Samples were divided into training (*n* = 110), validation (*n* = 28), and testing (*n* = 28) sets. A Gradient Boosting Classifier used seven study-derived features. (**H**) ROC curves from the testing set comparing the model with CRC-specific or pancancer biomarkers. In (D) to (F), data are presented as mean ± SEM. Unpaired two-tailed *t* test was used for (D) and (F). Two-way ANOVA followed by Tukey’s multiple comparisons test was used for (E). Created in BioRender. Lab, S. (2026) https://BioRender.com/m093dka.

## DISCUSSION

Our study delineated a previously uncharacterized pleiotropic signaling axis initiated by SLC39-mediated zinc influx that orchestrates immunosuppressive reprogramming in CRC. Using SLC transporter–centered multi-omics clustering, we identified a distinct patient subgroup aligned with the CMS2 molecular subtype, characterized by WNT activation, MSS, and an immune-desert phenotype. This CMS2-like archetype was characterized by overexpression of the SLC39 family, particularly SLC39A4 and SLC39A5, which drives zinc influx and triggers a transcriptional cascade culminating in epigenetic activation of CD24—a recently recognized “don’t eat me” signal analogous to CD47. We found that zinc functions as a cofactor that enhances binding of the CDX2 TF to a CD24 regulatory enhancer, promoting its overexpression. This is consistent with prior evidence that zinc activates CDX2 phosphorylation and activity via the phosphoinositide 3-kinase (PI3K)–CDX2 pathway to regulate downstream targets in intestinal epithelial cells (*60*). Multi-omics correlation, chromatin accessibility profiling, enhancer activity assessment, and three-dimensional (3D) protein-DNA docking analyses collectively established the direct transcriptional control of CD24 by this zinc-responsive TF. Functional validation in CMS3-type CRC cells, which lack zinc signature enrichment, demonstrated that the ectopic expression of SLC39A4 or SLC39A5 was sufficient to elevate intracellular zinc levels, activate CDX2, and induce CD24 expression at both the transcript and membrane-localized protein levels. Notably, the zinc-dependent overexpression of CD24 occurred independently of CD47, defining a discrete immunoevasive pathway in CMS2-like tumors. At the patient level, the SLC39-driven zinc influx state was robustly recapitulated and associated with significantly reduced progression-free and disease-free survival across more than 250 patients with TCGA-COAD, as well as resistance to ICIs in 26 patients with CRC, where elevated expression of “do not eat me” signals such as CD24 or CD47 has been reported to correlate with diminished ICI efficacy and primary resistance in colorectal and head and neck cancer (*61*, *62*). Extending our analysis to the TME, we found that CD24 overexpression in cancer cells facilitated immune-evasive communication via SIGLEC10-expressing myeloid cells. This interaction exhibited lineage-specific pleiotropy; the engagement of SIGLEC10 on moMCs and moDCs led to the repression of phagocytosis-related genes, whereas signaling in RTMCs favored adhesion-related pathways. Spatial and single-cell transcriptomic analyses further supported the idea that these pleiotropic CD24-SIGLEC10 interactions can define immunosuppressive niches at tumor borders in a subset of CRC tissues, where cancer–myeloid cell contact is spatially enriched. Furthermore, the CD24-SIGLEC10 interaction strength and downstream signaling modules correlated with nonresponsiveness to ICI therapy. Consistently, phagocytosis assays using moMC-like macrophages and CRC cells demonstrated that the overexpression of SLC39A4 or SLC39A5 suppressed macrophage-mediated engulfment, an effect reversed by CD24-SIGLEC10 blockade. To translate these mechanistic findings into a clinically applicable framework, we developed an ICI-response prediction model that integrates multicohort transcriptomic data. The model, trained on a seven-gene *SLC39*-*CD24*-*SIGLEC10* signature, achieved the highest predictive performance (AUC = 0.728) in an integrated ICI-treated CRC cohort, establishing a zinc-driven immunosuppressive program as a robust molecular predictor of the therapeutic response. Although clinical translation of this axis is still emerging, anti-SIGLEC10 antibodies, such as ONC-841, have recently entered early-phase clinical trials (*63*), highlighting the druggability of this checkpoint pathway, whereas CD24-targeting antibodies (e.g., IMM47) have demonstrated preclinical efficacy but remain at the investigational stage (*64*). These results confirmed that SLC39-mediated zinc influx establishes a therapeutically actionable axis linking metabolic reprogramming to innate immune suppression. Together, our findings identify SLC39-driven zinc influx as a central regulator of immunosuppressive plasticity in CMS2-like CRC and reveal a multimodal immune-evasion mechanism orchestrated through pleiotropic CD24-SIGLEC10 signaling.

SLC transporters have been broadly implicated in tumor metabolism and microenvironmental remodeling (*4*). However, their roles in CRC remain incompletely defined, particularly in the context of immune-desert or cold tumor states. Here, we delineated a mechanistic axis in which SLC39-driven zinc influx orchestrates an immunosuppressive program characteristic of the CMS2-like molecular subtype. This signaling cascade links metabolic reprogramming to immune evasion, establishing zinc-dependent transcriptional control as a critical determinant of tumor–immune cell interactions. Within this framework, CD24-SIGLEC10 emerges as a contextually prominent antiphagocytic pathway in SLC39-enriched CMS2-like tumors. Specifically, SLC39-mediated zinc influx should be viewed as an activity-modulating signal that amplifies CDX2-dependent CD24 expression in permissive CRC contexts rather than as a universal driver across all CRC cells. Although CD47-SIRPα signaling is a canonical “don’t eat me” pathway frequently up-regulated in CRC and known to potentiate adaptive immune priming upon blockade (*65*, *66*), our data show that SLC39-mediated zinc influx preferentially promotes CDX2-dependent *CD24* transcription without the concomitant up-regulation of CD47 expression. This nominates CD24-SIGLEC10 as a contextually prominent innate immune checkpoint candidate in SLC39-enriched, CMS2-like CRC, while highlighting the need for direct comparisons with CD47-SIRPα to define its relative dominance and therapeutic relevance (*23*). Our integrative analyses reveal that CD24-SIGLEC10 interactions extend beyond tumor-associated macrophages, engaging multiple myeloid subsets to enforce pleiotropic immunosuppression. Among these, SPP1^+^ moMCs sculpt hypoxic, fibroblast-enriched niches associated with poor prognosis (*67*, *68*), whereas CCR7^+^LAMP3^+^ moDCs acquire mregDC programs that dampen T cell priming (*69*). RTMCs activate adhesion and hypoxia-adaptive pathways. Collectively, these lineage-specific programs demonstrated that the zinc-dependent up-regulation of CD24 expression in tumor cells enables SIGLEC10 engagement across multiple myeloid compartments, thereby extending the canonical CD24-SIGLEC10 immune checkpoint into a multicellular suppressive circuit.

While our study delineates the mechanistic underpinnings of SLC39-driven zinc influx and CD24-mediated immune evasion in CMS2-like CRC, it also reveals the distinct regulatory architectures that underpin other archetypes. Archetypes 2 and 3, associated with the SLC22 and SLC35 families, respectively, exhibit immune and proliferative traits that are not attributable to zinc signaling yet converge on unique CMS subtypes. As shown in Fig. 1D, archetype 2 aligned with the CMS4 subtype and was characterized by epithelial-mesenchymal transition, angiogenesis, and the relative repression of cell-cycle pathways. Notably, this subtype is characterized by the epigenetic silencing of SLC22 transporters, such as SLC22A18 and SLC22A5, of which promoter hypermethylation enhances tumor cell invasion and survival (*70*, *71*). In contrast, archetype 3 recapitulates the features of CMS1 tumors that harbor MSI and mismatch repair deficiency. These tumors paradoxically couple defective DNA repair with proliferative advantages due to impaired TGF-β signaling (*72*, *73*). The aberrant methylation and suppression of *SLC35A3*, encoding a nucleotide-sugar transporter, has been implicated in compromised DNA repair fidelity and attenuated immune infiltration (*74*). Together, these findings suggest that CRC subtypes use divergent transporter circuits: SLC22 silencing supports mesenchymal transition in CMS4, SLC35A3 repression augments immune dysfunction in CMS1, and SLC39-mediated zinc signaling orchestrates immune escape in CMS2. This underscores the metabolic plasticity that stratifies CMS and reveals context-dependent vulnerabilities across CRC archetypes (*75*). At the same time, caution is warranted when assigning metabolic characteristics to molecular subtypes through rigid or discrete classification, because the clinical utility of CMS stratification remains limited by substantial intratumoral heterogeneity (*28*, *76*). Increasing evidence indicates that biological variability within established CMS groups reflects differences in the cellular composition and spatial architecture (*25*), suggesting the presence of distinct subgroups with unique clinical and biological properties (*77*). These considerations underscore the need for refined cell-resolved frameworks that integrate metabolic programs with the tumor architecture to improve subtype definition and therapeutic stratification.

Acknowledging that our study did not use mass spectrometry–based metabolic flux assays to directly quantify metabolite exchange, we sought to overcome this gap by leveraging multi-omics integration, pathway-level analyses, and reaction-level flux modeling. Recent high-impact studies have demonstrated that transcriptomic and proteogenomic profiling can be used to robustly infer tumor metabolic rewiring, such as glycolytic activation in MSI-high colorectal tumors (*78*) and oxidative phosphorylation imbalances in clear cell renal cell carcinoma (*79*). Similarly, integrative omics of thyroid cancers revealed subtype-specific TCA cycle and one-carbon metabolism programs with elevated transporters and enzymes (*80*), whereas the proteogenomic stratification of non–small cell lung cancer uncovered a distinct metabolic subtype with heightened glycolytic and bioenergetic activity (*22*). Moreover, spatial multi-omics analysis in gastric cancer has shown that correlating metabolite distributions with gene expression can resolve cell type–specific metabolic cross-talk in the TME (*81*). Beyond conventional enrichment-based methods, the metabolic flux model (*24*) applied in this study was validated by its ability to recapitulate lipid-induced shifts toward serine-methionine metabolism in breast epithelial subpopulations, revealing redox-coupled metabolic plasticity that mirrors cancer-associated reprogramming dynamics (*82*). Together, these references underscore that, although direct metabolite tracing is not feasible, our use of multi-omics data and pathway inference represents a rigorous and widely validated strategy for dissecting tumor metabolic states. In bulk transcriptomic analyses of CRC, the predominance of malignant epithelial cells often masks immune-derived signals, limiting the sensitivity of projecting moDC, moMC, or RTMC signatures for prognostic stratification or subtype classification. The high tumor purity observed in TCGA and similar cohorts skewed the expression profiles toward tumor-intrinsic programs, which tended to show stronger correlations with clinical outcomes than the immune-related signatures (*83*, *84*). Notably, molecular subtypes initially interpreted as mesenchymal tumor cell states were later attributed predominantly to stromal and immune cell expression, revealing how shifts in the cellular composition can confound transcriptomic interpretations (*85*). Our spatial transcriptomic analysis also revealed a CD24-SIGLEC10–enriched niche architecture in two of four CRC tissues, indicating patient-level heterogeneity in the spatial organization of this immunosuppressive program. Nevertheless, patient-level bulk transcriptomic analyses showed that tumors with concurrent enrichment of SIGLEC10-expressing myeloid populations, including moMCs and RTMCs, exhibited the strongest predicted CD24-SIGLEC10 interaction potential and coordinated activation of downstream immune-evasive and adhesion-related programs. These findings suggest that although the spatial organization of the CD24-SIGLEC10 niche is heterogeneous, the underlying signaling axis is robustly activated in a defined subset of patients with CRC. Thus, the weaker associations observed for immune cell signatures in bulk CRC datasets likely reflect tumor cell dominance and interpatient heterogeneity, rather than a lack of biological relevance.

This study provides the most comprehensive evidence linking metabolic reprogramming via SLC transporters to tumor microenvironmental phenotypes, including canonical molecular subtypes, in CRC. Specifically, we identified a CMS2-like patient subgroup in which SLC39-mediated zinc influx drives CD24 overexpression via epigenetic enhancer activation in cancer cells. This zinc-dependent signaling cascade establishes pleiotropic cell-cell interactions with myeloid-lineage cells—moDCs, moMCs, and RTMCs—leading to immune-evasive “don’t eat me” signaling and adhesion remodeling. Notably, this immunosuppressive mechanism is independent of the canonical CD47 axis and is enriched in CMS2-like tumors, which are frequently associated with *SLC39* copy number gains. To extend these findings to clinical translation, we developed an ICI-response-prediction model that integrates multicohort transcriptomic data. The model, trained on a seven-gene zinc-CD24-SIGLEC10 signature, achieved superior accuracy in predicting the therapeutic response, highlighting the clinical relevance of this zinc-driven immunosuppressive program. Our findings suggest that therapeutic strategies targeting the SLC39–zinc influx–CD24 axis, including the blockade of CD24-SIGLEC10 interactions, may reverse immune exclusion in this CRC subtype. Given the lineage-specific engagement of SIGLEC10^+^ myeloid populations in CMS2-like tumors, engineering chimeric antigen receptor–based interventions targeting these myeloid subsets is a promising direction for precision immunotherapy.

## MATERIALS AND METHODS

### Patient sample collection and multi-omics profiling

The primary dataset used for this study was obtained from TCGA, specifically focusing on CRC samples. A total of 258 CRC samples were selected, encompassing multi-omics data including transcriptomics, mutations, CNV, and DNA methylation. These multi-omics profiles were retrieved from Xena Browser (https://xenabrowser.net/), which provides access to publicly available TCGA datasets. These multi-omics data were used to investigate the molecular landscape of CRC. For data validation and additional analyses, we used data from the CPTAC, which contains proteomic and phosphoproteomic profiles of CRC samples. A total of 109 CRC samples were included in this validation cohort. The CPTAC data were retrieved from Linkedomics (https://linkedomics.org/), which provides integrative analyses of omics datasets. These data were used to cross-validate findings from the TCGA dataset and further refine the molecular characterization of CRC. Clinical information for the TCGA cohort is provided in table S1, and corresponding information for the CPTAC cohort is available in table S2.

### Data integration and gene selection

To investigate metabolic dependencies in CRC, we focused on SLC, which regulate the import and export of metabolites, as well as cancer-specific oncogenic driver genes. We retained only the genes annotated as part of the SLC family or as known oncogenes in CRC. The list of SLC genes was curated by excluding pseudogenes and antisense transcripts to ensure biological relevance and transcriptional activity. For oncogenic driver genes, we used the OncoVar database, which provides curated lists of cancer type–specific driver genes along with a scoring system reflecting their pathogenic potential. Genes annotated for colorectal adenocarcinoma (COAD) with a driver level of 4 (i.e., “pathogenic,” score ≥20) were selected for analysis. In addition, to increase the robustness of the selected oncogenes, we further filtered for genes with a total score ≥3, representing the number of supporting databases in which the gene was identified as a driver. This filtering strategy ensured that only metabolically and oncogenically relevant genes were retained for subsequent integrative multi-omics analyses.

### Dimensionality reduction and multi-omics integration

To integrate the multiple layers of omics data and identify latent molecular features associated with CRC, we applied MOFA, a statistical framework designed for unsupervised integration of multi-omics datasets. MOFA enables extraction of low-dimensional latent factors that capture sources of variation shared across or specific to individual omics layers. We constructed a MOFA model using five omics layers: transcriptomics, somatic mutations, CNVs, DNA promoter methylation, and DNA gene body methylation. Given the nature of the data, we specified “Gaussian” likelihoods for continuous data types (transcriptomics, CNVs, and methylation), and “Bernoulli” likelihood for the mutation matrix, which was binarized to represent the presence or absence of nonsynonymous variants in each gene. The model was trained using the following parameters: The number of latent factors was set to 10, the convergence mode was set to “slow” to ensure a thorough exploration of the posterior space, and maxiter was set to 10,000 to allow sufficient iterations for convergence. This dimensionality reduction provided a shared latent representation of the dataset, which was used for downstream clustering, module discovery, and association analyses with clinical or immunological features.

### Archetypal analysis using ParetoTI

To identify representative molecular states, archetypal analysis was performed using the ParetoTI R package, which geometrically infers extremal points (archetypes) from input dimensional space. As input, the top four latent factors derived from MOFA were selected based on the proportion of explained variance. All six pairwise combinations of these four factors were evaluated using the *t*-ratio metric, which quantifies the fit between the polytope and convex hull of the data. The factor pair with the highest *t*-ratio (factor 1 and factor 4) was selected for downstream modeling. To functionally annotate the selected factors, gene set variation analysis (GSVA) was performed using metabolic pathway gene sets curated from Gene Ontology (GO: biological process category, filtered for terms containing “metabolic process”) and KEGG (metabolism category). Enrichment scores were computed for each sample and correlated with factor values to support interpretation of the molecular programs captured by the selected latent dimensions.

### Archetype assignment and multi-omics characterization

Samples were assigned to one of three archetypes (Arc1, Arc2, and Arc3) based on proximity to polytope vertices inferred by ParetoTI using MOFA-derived latent factors. To annotate biological relevance, CMS were inferred using the CMScaller R package. Molecular characteristics of each archetype were evaluated by analyzing enrichment patterns across multi-omics layers. Specifically, transcriptomic, promoter methylation, and gene body methylation profiles of SLC genes were compared across archetypes. GSVA was performed using curated pathway collections from GO, KEGG, WikiPathways, Reactome, and MSigDB Hallmark gene sets, focusing on the identification of pathways enriched in each archetype. To assess TME differences, xCell was used to infer immune and stromal cell–type enrichment scores from bulk transcriptomic data. Archetype-specific correlations with TME composition were calculated using Pearson correlation to evaluate immune landscape variation across subtypes.

### Zinc pathway scoring and flux estimation

To evaluate zinc-associated metabolic activity, pathway scores were computed based on the MAR05989 zinc transport reaction defined in the Human-GEM (the generic genome-scale metabolic model of *Homo sapiens*) (*86*). The associated gene set included SLC7A6, SLC39A14, SLC11A2, SLC39A8, SLC39A5, SLC39A6, SLC39A3, SLC39A1, SLC39A4, SLC39A2, and SLC39A10. GSVA was used to compute zinc pathway scores across bulk transcriptomic samples. In addition, zinc flux scores—representing extracellular to intracellular zinc import—were inferred using the COMPASS algorithm (*24*), based on the same MAR05989 reaction.

### Inference of protein activity using VIPER

To infer protein activity from transcriptomic data, we applied the VIPER algorithm (*27*). VIPER estimates relative protein activity by evaluating the expression of their transcriptional targets (regulons), allowing protein-level functional inference from RNA expression profiles. For this analysis, we used a COAD-specific regulatory network generated by ARACNe (*87*) as the reference regulon input.

### Identification of CD24 enhancer region and TF binding motifs

Putative enhancer elements regulating CD24 were identified using the GeneHancer database (GH06J106781). Open chromatin accessibility at this region was confirmed using ATAC-seq data from 25 COAD samples (*33*). To investigate enhancer activity specific to the archetype 1, chromatin accessibility signals were compared across archetype-classified samples. Topological organization of the enhancer-promoter interaction was assessed using Hi-C data via the 3D Genome Browser (http://3dgenome.org), which revealed that both CD24 and GH06J106781 reside within the same TAD, supporting the potential regulatory interaction. To identify TFs that potentially bind to the enhancer, motif enrichment analysis was performed using the TFBSTools R package in conjunction with the JASPAR2022 motif database (*88*). The enhancer sequence was scanned for TF binding motifs using position weight matrices (PWMs) from JASPAR, and matches were identified based on a minimum score threshold of 80% relative to the minimal PWM score. For each candidate binding site, associated *P* values and matched binding sequences were computed using searchSeq(), and high-confidence hits were retained for downstream structural modeling. Clinical information for the scATAC-seq cohort is summarized in table S3.

### Assessment of enhancer histone marks using ENCODE ChIP-seq data

To annotate enhancer activity and validate motif accessibility, publicly available ChIP-seq datasets from the ENCODE (*31*) project were used. Specifically, we downloaded ChIP-seq data for histone modifications in CRC cell lines, including H3K27ac (ENCSR571QQB), H3K36me3 (ENCSR000DQK), and H3K4me1 (ENCSR061UOM). These datasets were used to assess the chromatin landscape of candidate enhancer regions identified through motif analysis, providing epigenomic evidence for TF binding and regulatory activity in CRC contexts.

### Inference of kinase activities from phosphoproteomic profiles

We examined raw intensity values of CDX2 phospho-S176 across human tumor tissues using publicly available phosphoproteomic data from PRIDE under the accession PXD012174. To estimate kinase activity from phosphoproteomic data, we applied the KSTAR (*89*) package to CRC samples from the CPTAC-COAD cohort (*n* = 84). Phosphorylation sites were first mapped to known kinase–substrate relationships using the KinPred ontology integrated in KSTAR. Activity scores were then calculated for each sample by evaluating the likelihood that the observed phosphosites were nonrandomly enriched within kinase-specific substrate networks. Null distributions were generated by random sampling of phosphosites with matched study bias, and statistical significance was assessed using the Mann-Whitney *U* test. Final activity scores were normalized against the null model and used for downstream analyses including correlation with transcriptomic programs.

### Structural modeling and protein-DNA docking of CDX2

The 3D structures of CDX2 and its phosphorylated form at serine-176 (CDX2-pS176) were predicted using AlphaFold3 (*38*). To evaluate the physical interactions between the CD24 enhancer sequence and distinct CDX2 conformations, DNA-protein docking analysis was performed using the HDOCK (*39*) web server. Docking was conducted separately for unmodified CDX2 and CDX2-pS176 using the enhancer-matched binding motif sequences defined from prior motif analysis.

### Single-cell RNA-seq data processing and clustering

Tumor-derived single-cell RNA-seq data were obtained from the Synapse dataset (accession: syn26844071). Only cells annotated as originating from tumor tissue were included in the analysis. Quality control filtering was applied to retain cells with more than 200 detected genes, fewer than 100,000 total counts, and less than 3% mitochondrial gene content. Genes expressed in fewer than three cells were removed. The processed data were normalized and scaled, and 2000 highly variable genes were selected for downstream analysis. Principal components analysis (PCA) was performed, and the first 10 dimensions were used for clustering (resolution = 0.5) and UMAP-based visualization. All analyses were conducted using Seurat v5.2.1. Clinical information for this cohort is summarized in table S4.

### Inference of CNVs from single-cell transcriptomes

To infer large-scale CNV at single-cell resolution, we applied the inferCNV (*26*) R package (v1.14.2) to the processed scRNA-seq data. Tumor epithelial cells, derived from scRNA-seq of matched normal colorectal tissue, were analyzed using adjacent normal epithelial cells as reference. Genes with an average expression below 0.1 were filtered out, and denoising steps were applied to reduce technical noise and improve CNV signal clarity.

### Metacell construction using SEACells

To overcome technical noise and data sparsity inherent in scRNA-seq data, we applied the SEACells (*40*) algorithm to identify robust transcriptional metacell states. A total of 1700 SEACells were initialized across the dataset, with metacell resolution controlled by setting convergence_epsilon = 1 × 10^−5^, min_iter = 20, and max_iter = 30. PCA was used to construct the cell-cell similarity kernel. The algorithm was run in default mode with adaptive fitting of the SEACells model to stabilize local transcriptomic structure.

### Trajectory inference using Palantir and CellRank

Trajectory analysis was performed on single-cell RNA-seq data restricted to cancer cells. Diffusion-based pseudotemporal ordering was computed using the Palantir (*42*) algorithm. First, diffusion maps were generated using palantir.utils.run_diffusion_maps with 10 components and PCA coordinates derived from UMAP embedding. Palantir was then run with 500 waypoints and manually defined terminal states using palantir.core.run_palantir. The resulting pseudotime values were used as input for CellRank (*43*) to compute lineage probabilities. CellRank integrated Palantir pseudotime and transcriptomic similarity into a combined kernel to infer fate probabilities and terminal states. These were used to model cell-state transitions and identify lineage-associated gene programs.

### Trajectory-based signature derivation and TCGA deconvolution

Following trajectory inference using Palantir and CellRank, three terminal states (terminals 1 to 3) were identified from the pseudotemporal progression of cancer cells. To characterize these terminal states, we constructed gene expression signature matrices for each terminal state using the buildSignatureMatrixMAST function from the DWLS (Digital Weighted Least Squares) R package. These trajectory-derived signatures were subsequently applied to bulk TCGA transcriptomic data using CIBERSORT (*90*) to infer the enrichment of each terminal program across patient samples.

### Pathway enrichment analysis in single-cell and spatial transcriptomics

Pathway-level enrichment analyses at both single-cell and spatial transcriptomic resolution were performed using the escape R package, which facilitates integration of gene expression data with gene set enrichment analysis. The tool supports enrichment computation from Seurat and SingleCellExperiment objects and enables access to the Molecular Signature Database (MSigDB v7.0), including Hallmark and other curated gene sets. Enrichment scores were computed for selected gene sets of interest, and pathway activity was assessed across individual cells or spatial regions.

### Survival analysis based on omics-derived signatures

Progression-free interval and disease-free interval analyses were conducted using TCGA CRC samples. For each molecular signature of interest, patients were stratified into high and low groups based on optimal cutpoints. These cutpoints were computed using the maxstat R package, which identifies the most statistically significant threshold via maximally selected rank statistics. Kaplan-Meier survival curves were generated, and group differences were assessed using the log-rank test.

### Single-cell RNA-seq dataset from ICI-treated CRC

Single-cell transcriptomic data from ICI-treated CRC was obtained from the publicly available dataset SCP2079, which corresponds to the study “Combined PD-1, BRAF and MEK inhibition in BRAFV600E colorectal cancer.” For this study, we specifically used the pretreatment samples to analyze the baseline immune and tumor microenvironmental states before therapy. Clinical information for this cohort is summarized in table S5.

### Construction of response- and PFS-associated gene networks

To investigate functional gene connectivity associated with treatment response and PFS at single-cell resolution, we constructed cell type–specific gene interaction networks using the scHumanNet (*45*) framework. This method uses the SCINET algorithm in conjunction with the HumanNet v3 interactome to infer context-specific gene networks from single-cell RNA-seq data. SCINET incorporates imputation, transformation, and normalization steps via the ACTIONet package to robustly model gene-gene interactions in sparse single-cell data. Subsampling was applied per cluster to stabilize interaction inference across cell types. Response-specific and PFS-specific networks were generated separately. For downstream network characterization, we computed connectivity metrics for marker genes identified in each terminal state, including degree centrality, eigenvector centrality, and PageRank scores. These metrics were used to assess the relative influence of terminal-associated genes within the constructed networks, providing insights into their functional roles in response and survival-associated pathways.

### Cell-cell interaction analysis using NICHES in single-cell and spatial transcriptomics

To investigate intercellular communication across tumor and stromal compartments, we used the NICHES (*91*) framework to compute cell-cell interactions based on known ligand-receptor relationships. In the single-cell RNA-seq dataset, cancer cells were designated as ligand-sending cells, and pairwise interactions with all other cell types were calculated at the individual cell level. For each recipient cell type, interaction scores were derived across all ligand-receptor pairs, and top-ranked interactions were identified using the Seurat FindMarkers function. In parallel, spatial transcriptomics data were also processed using NICHES, enabling spatially contextualized analysis of cell-cell signaling. For these data, interactions were assessed using spatially colocalized expression patterns, allowing for visualization and inference of neighborhood-level communication.

### Ligand-target interaction analysis using NicheNet

To further characterize intercellular communication pathways involving cancer-derived CD24 signaling, we used NicheNet (*92*), a computational framework that integrates ligand-receptor binding, intracellular signaling, and transcriptional regulation into a comprehensive ligand-target prior model. This model provides a quantitative matrix describing the potential regulatory influence of each ligand on downstream target genes based on integrated biological knowledge. As input, cell type–specific marker genes were identified from single-cell RNA-seq data using Seurat’s FindMarkers function. We then filtered these marker genes using NicheNet’s ligand-target matrix to retain only those with a regulatory score of ≥0.0005 for CD24, the ligand of interest. The ligand activity score in NicheNet reflects how well a given ligand is predicted to regulate its downstream target genes, based on prior knowledge from curated signaling and gene regulatory networks. To gain insight into the biological functions of the identified target genes, we performed pathway enrichment analysis using the enrichGO function from the clusterProfiler R package. This allowed us to identify GO terms and biological processes significantly associated with CD24-regulated transcriptional programs in each recipient cell population.

### Ligand-receptor scoring in bulk RNA-seq data

Ligand-receptor interaction scores were calculated from bulk RNA-seq data using the decoupleR (*51*) package. Ligand-receptor pairs were obtained from the LIANA (*93*) resource and reformatted into gene sets, with each pair treated as a functional unit. Enrichment scores were computed using a weighted mean-based approach. All analyses were conducted using default parameters.

### Spatial transcriptomics data acquisition and preprocessing

Spatial transcriptomics data were obtained from the publicly available dataset GSE225857, which includes FFPE tissue sections from four samples from patients with CRC. The dataset was generated using the 10x Genomics Visium Spatial Gene Expression platform for FFPE tissues, and sequencing was performed on the Illumina NovaSeq 6000 system. For this study, we used the preprocessed UMI count matrices and accompanying hematoxylin and eosin (H&E)–stained tissue images provided with the dataset. Spatial transcriptomic analyses were carried out using Seurat (v5.2.1) and the SPATA2 (*48*) package in R. Analyses included quality control filtering, normalization, dimensional reduction, clustering, and spatial feature extraction. Clinical information for this cohort is summarized in table S6.

### Cell-type annotation of spatial transcriptomics data

Cell-type deconvolution of the spatial transcriptomics dataset was performed using SPOTlight (*47*), which enables reference-based mapping of spatial spots to single-cell identities. A reference atlas was generated from the annotated single-cell RNA-seq dataset of tumor-derived epithelial and stromal cells obtained from the EGAC00001002526 cohort, which had undergone rigorous quality control, normalization, and clustering as described above. SPOTlight was then applied to infer the proportional representation of each cell type within individual spatial transcriptomic spots.

### Spatial enrichment analysis of CD24-SIGLEC10 interaction score

To delineate regions with elevated CD24-SIGLEC10 interaction within the spatial transcriptomic landscape, we calculated interaction scores across all spatial spots using the createNumericAnnotations function from the SPATA2 package. Spots with scores exceeding the 80th percentile—derived from the overall distribution of CD24-SIGLEC10 interaction values—were designated as enriched regions. Spatial coherence of these regions was further refined using inner_borders = TRUE and a concavity parameter of 1.5, enabling precise identification of TME niches with potentially high CD24-SIGLEC10 signaling activity.

### Spatial niche identification and multilevel signaling landscape analysis

To identify spatially distinct tumor niches and characterize their associated signaling ecosystems, clustering was performed on the spatial transcriptomic data using Seurat. After setting the default assay to SCT (single-cell transform), unsupervised clustering was conducted using FindClusters, followed by UMAP projection for spatial visualization. For each resulting niche, key signaling axis scores—including those reflecting tumor-intrinsic pathways, intercellular communication, and downstream responses in recipient cell populations—were averaged and quantile normalized to a 0 to 1 range. To identify niche-specific signaling programs, two-sided *t* tests were conducted comparing each niche to all others, with corresponding *P* values used to determine statistically enriched signaling states.

### Projection of single-cell–derived macrophage signatures onto bulk transcriptomes

To investigate the functional distribution of macrophage subtypes at the bulk level, we derived marker gene signatures for RTMCs and moMCs/moDCs from single-cell RNA-seq data. The top 50 nonoverlapping marker genes were identified for each subtype to ensure subtype specificity. These gene sets were then used to calculate GSVA enrichment scores for each TCGA-COAD sample, yielding sample-level RTMC and moMC/moDC scores. The samples were projected onto a two-dimensional space defined by these scores, and unsupervised clustering was performed using *k*-means (*k* = 4) to stratify the samples into four groups with distinct macrophage-related immunological features.

### Cell lines and cell culture

All used cell lines were of human origin and tested for mycoplasma contamination. LS174T and HEK293T cells were kindly provided by Y.-H. Lim and F. McCormick, respectively. HCT116 (10247), NCIH508 (10253), and THP-1 (40202) cells were purchased from the Korean Cell Line Bank (KCLB; Seoul, South Korea). LS174T and HEK293T cells were maintained in Dulbecco’s modified Eagle’s medium (DMEM; Welgene), and HCT116, NCIH508, and THP-1 cells were maintained in Roswell Park Memorial Institute (RPMI; Welgene) medium. Both media were supplemented with 10% fetal bovine serum (FBS, Gibco, 12483020) and 1% penicillin-streptomycin (Hyclone, SV30010). All cells were cultured in a humidified incubator at 37°C with 5% CO_2_.

### Lentiviral constructs and transduction

The CRISPR-dCas9 Synergistic Activation Mediator (SAM) system was used as a CRISPR activation (CRISPRa) tool to enhance gene expression. This system comprised two plasmids: lentiMPHv2 (Addgene, 89308) and lentiSAMv2 (Addgene, 75112). Single guide RNAs (sgRNAs) were designed using the CRISPICK and CHOPCHOP platforms and synthesized by Bionics (Korea). sgRNA sequences were cloned into the lentiSAMv2 backbone according to the protocol provided by Addgene (*94*). Briefly, lentiSAMv2 was digested with Esp 3I (Enzynomics, R116S), and annealed oligonucleotides were ligated into the digested vector. shRNAs were cloned into LT3GEPIR (Addgene, 111177) from which green fluorescent protein (GFP) had been excised. Briefly, the 97-mer oligonucleotides were designed using Splash platform and synthesized by Bionics, followed by PCR amplification using the primers LT3GEIRP-fw (ACTTCGACTTCTTAACCCAACAGAAGGCTCGAGAAGGTATATTGCTGTTGACAGTGAGCG) and LT3GEIRP-rev (TTTAGTAAACAAGATAATTGCTCGAATTCTAGCCCCTTGAAGTCCGAGGCAGTAGGCA). Final vectors were obtained by Eco RI (Enzynomics, R002S)/Xho I (Enzynomics, R007S) cloning of the purified PCR products into recipient vectors. Positive constructs were verified by Sanger sequencing. For lentiviral production, HEK293T cells were seeded in six-well plates and cultured overnight to reach 70 to 80% confluence. Cells were transfected with a plasmid mixture of pMD2.G (Addgene, 12259), psPAX2 (Addgene, 12260), and transfer plasmids using polyethylenimine in serum-free DMEM. After 6 hours, the medium was replaced with DMEM supplemented with 10% FBS and 1% penicillin-streptomycin. The viral supernatant was harvested 48 hours later, filtered through a 0.45-μm filter, and used for transduction. lentiMPHv2 or LT3GEPIR-shRNA infection was performed for 48 hours, followed by hygromycin or puromycin selection, respectively. For gene activation, lentiMPHv2 cells were infected with lentiSAMv2-sgRNA for 48 hours and selected with blasticidin. The sgRNA-97mer sequences (5′-3′) were as follows: sgCDX2, GAGGTTAAAGTGCACCAGGT; sgSLC39A4, GGTTGTCCAGGGCCAGACTG; sgSLC39A5, GGTGGGGTGTCCCTAGAAGG; shSLC39A5, TGCTGTTGACAGTGAGCGAGAGCAATAGGATTTTAATAAATAGTGAAGCCACAGATGTATTTATTAAAATCCTATTGCTCCTGCCTACTGCCTCGGA.

### Cas9 RNP-mediated CDX2 knockout

crRNA sequence was designed using CHOPCHOP platform, and gRNA complexes were formed by annealing crRNAs and tracrRNAs at 95°C for 5 min. Recombinant Cas9-GFP (Integrated DNA Technologies (IDT)) was incubated with gRNAs to form RNP complexes. NCIH508 cells were resuspended in Lonza SF buffer and electroporated with the RNP complexes and Alt-R Cas9 electroporation enhancer (IDT) using Nucleofector (Lonza, pulse code FF137). Monoclonal cell lines were isolated via the serial dilution method. The CDX2 crRNA (5′-3′) sequence was as follows: GGACTACGGCGGTTACCACG.

### Real-time qPCR

Total RNA was isolated from cultured cells using RiboEx (GeneAll, 301) and quantified with a NanoDrop spectrophotometer (Thermo Fisher Scientific). Complementary DNA (cDNA) was synthesized from 2.5 μg of total RNA using the RevertAid First Strand cDNA Synthesis Kit (Thermo Fisher Scientific, K1621) in a total volume of 10 μl. Quantitative PCR was performed on either a StepOnePlus or QuantStudio 6 Flex Real-Time PCR System (Applied Biosystems) using SYBR Green reagents (Enzynomics, RT500 or RT501). Gene expression levels were normalized to β-actin, and relative expression was calculated using the ΔΔCt method. All primer sequences used in this study are listed in table S7.

### Western blot and antibodies

Cells were lysed in RIPA buffer supplemented with protease and phosphatase inhibitor cocktails. Protein concentrations were determined using a Bradford assay. Equal amounts of protein (30 to 40 μg) were separated by SDS–polyacrylamide gel electrophoresis, transferred to polyvinylidene difluoride membranes, and blocked with 3% bovine serum albumin (BSA) in TBST. Membranes were incubated with primary antibodies overnight at 4°C, followed by horseradish peroxidase–conjugated secondary antibodies for 1 hour at room temperature. Protein bands were detected using an ECL detection kit (Thermo Fisher Scientific, 32106 or GLPBio, GK10008) and imaged with a UVP ChemStudio system (Analytik Jena). Primary antibodies were as follows: anti-CD24 [clone SN3, Santa Cruz Biotechnology, SC-19585, AB_626989; Western blot (WB) 1:1000; flow cytometry and phagocytosis assay 10 μg ml^−1^] and anti-CDX2 [clone D11D10, Cell Signaling Technology, 12306, AB_2797879; WB 1:1000; immunofluorescence (IF) 1:400]. Secondary antibodies included Goat anti-Mouse IgG (H+L) Cross-Adsorbed Secondary Antibody, Alexa Fluor 488 (Invitrogen, A-11001, AB_2534069; flow cytometry 1:500) and Goat anti-Rabbit IgG (H+L) Cross-Adsorbed Secondary Antibody, Alexa Fluor 594 (Invitrogen, A-11012, AB_2534079; IF 1:500).

### Immunofluorescence

Cells were seeded on coverslips and fixed with 4% paraformaldehyde for 15 min at room temperature. Fixed cells were washed three times with DPBS and permeabilized with 0.1% Triton X-100 for 10 min at room temperature, followed by blocking in 3% BSA in DPBS for 1 hour at room temperature. Cells were then incubated with primary antibodies diluted in 1% BSA in DPBS at 4°C overnight. After washing with DPBS, cells were incubated with fluorescently labeled secondary antibodies for 1 hour at room temperature. Nuclei were counterstained with DAPI, and coverslips were mounted using Fluoromount Aqueous Mounting Medium (Sigma). Images were acquired using an ECLIPSE Ts2-FL microscope (Nikon).

### Flow cytometry

Flow cytometric analyses were performed to measure intracellular Zn^2+^ levels and membrane CD24 expression. For intracellular Zn^2+^ quantification, cells were harvested by trypsinization and incubated with 1 μM FluoZin-3 (Invitrogen, F24195) for 30 min at 37°C. Cells were then washed twice with DPBS and further incubated for 30 min at 37°C to allow de-esterification. For analysis of membrane CD24 expression, cells were detached using Accutase (Millipore, SCR005), incubated with 10 μg ml^−1^ anti-CD24 antibody in FACS buffer (0.5% BSA in DPBS) for 1 hour on ice, washed twice with ice-cold FACS buffer, and then incubated with Alexa Fluor 488–conjugated secondary antibody for 30 min on ice before flow cytometric analysis. All samples were analyzed using an Attune NxT flow cytometer (Thermo Fisher Scientific) with excitation/emission, 488/520 nm. Data were analyzed using FlowJo software (v10, BD Biosciences), and MFI values were calculated from the fluorescence signals obtained.

### Phagocytosis assay

THP-1 cells were differentiated into macrophages by incubation with 100 ng ml^−1^ phorbol 12-myristate 13-acetate (GLPBio, GN10444) for 48 hours. Macrophages were polarized to the moMC phenotype with interferon-γ (20 ng ml^−1;^ Enzynomics, C006) and lipopolysaccharide (100 ng ml^−1;^ Sigma-Aldrich, L2880), or to the RTMC phenotype with interleukin-4 (IL-4; 20 ng ml^−1^; Enzynomics, C008) and IL-13 (20 ng ml^−1^; Enzynomics, C009) for 48 hours. A total of 2 × 10^5^ GFP^+^ THP-1 cells were differentiated into moMC-like macrophages on 18-mm coverslips in 12-well plates. LS174T cells were detached using Accutase, labeled with pHrodo Red succinimidyl ester (Thermo Fisher Scientific, P36600) at a 1:30,000 dilution in PBS for 1 hour at 37°C, and washed twice with DMEM supplemented with 10% FBS and 1% penicillin-streptomycin. Labeled LS174T cells (1 × 10^6^ per well) were added to macrophages in serum-free RPMI with anti-CD24 antibody (10 μg ml^−1^; clone SN3; Santa Cruz Biotechnology); an isotype control antibody (clone MOPC-21, BioXcell, BE0083, AB_1107784) was used at the same concentration. After 2 hours of coculture at 37°C, cells were washed five times with serum-free RPMI and imaged using a fluorescence microscope (ECLIPSE Ts2-FL, Nikon). Phagocytosis was quantified as the ratio of pHrodo Red+ GFP^+^ THP-1 macrophages.

### Statistical analysis in experimental validations

Statistical analyses were performed using GraphPad Prism version 6 (GraphPad Software). All experiments were performed in at least three independent biological replicates. Data are presented as mean ± SEM. For comparisons between two groups, exact *P* values were calculated using unpaired two-tailed *t* tests. For comparisons among more than two groups, *P* values were determined using either one-way or two-way analysis of variance (ANOVA), as appropriate, followed by Tukey’s multiple comparisons test. Data distribution was assumed to be normal, but this was not formally tested. Statistical significance was defined as *P* < 0.05.

### Constructing a predictive model for ICI response in CRC

To develop a predictive model of ICI response in CRC, we integrated five publicly available ICI-treated CRC transcriptomic cohorts (MSS1: *n* = 9, MSS2: *n* = 34, MSS3: *n* = 12, MSI1: *n* = 70, MSI2: *n* = 41), totaling 166 samples. Batch effects were removed using the removeBatchEffect function from the limma R package. The integrated dataset was randomly partitioned into a modeling set (*n* = 138) and an independent testing set (*n* = 28). The modeling set was further split into a training set (*n* = 110) and validation set (*n* = 28). Model construction was performed using the PyCaret machine learning framework in Python. PyCaret was used to automate the comparison of multiple classification algorithms under standardized settings (fold = 5, fold_strategy = StratifiedKFold, session_id = 2025). Among the tested models, the Gradient Boosting Classifier demonstrated the highest predictive performance (AUC = 0.7262) and was selected for final deployment. Other top-performing models included AdaBoost Classifier (AUC = 0.6735), Extra Trees Classifier (AUC = 0.6515), Extreme Gradient Boosting (AUC = 0.6314), and Random Forest Classifier (AUC = 0.6263). The final model was trained using seven biologically informed features—*SLC39A2*, *SLC39A4*, *SLC39A5*, *SLC39A10*, *CDX2*, *CD24*, and *SIGLEC10*—identified in this study as key regulators of zinc transport and immune evasion in CRC. Model performance was evaluated using the area under the receiver operating characteristic curve. To benchmark our model, we trained and evaluated additional Gradient Boosting Classifier using previously published gene sets known to be associated with ICI response in either pancancer or CRC-specific contexts (*52*–*59*). Each comparator model was trained using the same 138-sample modeling set and evaluated on the 28-sample testing set to assess generalizability. To ensure balanced representation of both clinical cohorts and response classes, each of the five ICI-treated CRC datasets (MSI1, MSI2, MSS1, MSS2, and MSS3) was independently split into modeling (85%) and external testing (15%) sets. Splitting was performed separately per cohort to maintain cohort-wise sample proportions in both subsets. As a result, the modeling set (*n* = 138) and external testing set (*n* = 28) preserved the original composition of the five cohorts: MSI1 (42.75% versus 39.29%), MSI2 (24.64% versus 25.00%), MSS1 (5.07% versus 7.14%), MSS2 (20.29% versus 21.43%), and MSS3 (7.25% versus 7.14%). Response class distribution was also maintained, with responders (R) comprising 62.32% of the modeling set and 53.57% of the testing set, and nonresponders (NR) comprising 37.68 and 46.43%, respectively. Clinical information for this cohort is summarized in table S8 and corresponding biomarker gene sets for benchmarking are summarized in table S9.
