# Supplementary material for: SLC39-driven zinc influx orchestrates pleiotropic tumor–immune system cross-talk to establish immune suppression in colorectal cancer
Source: Sci Adv. 2026 Oct 7;12(41):eaeh2362. doi: 10.1126/sciadv.aeh2362 (PMC13644755; doi:10.1126/sciadv.aeh2362)
Supplement: Supplementary file 1 [file sciadv.aeh2362_sm.pdf]

Science Advances
AAAS

Supplementary Materials for

SLC39-driven zinc influx orchestrates pleiotropic tumor–immune system cross-talk to establish immune suppression in colorectal cancer

Seok June Hong *et al.*

Corresponding author: Sung Eun Kim, sek19@korea.ac.kr; Kwoneel Kim, kwoneelkim@gmail.com

Sci. Adv. **12**, eaeh2362 (2026)
DOI: 10.1126/sciadv.aeh2362

The PDF file includes:

Figs. S1 to S7
Legends for tables S1 to S9
Legends for data S1 and S2

Other Supplementary Material for this manuscript includes the following:

Tables S1 to S9
Data S1 and S2

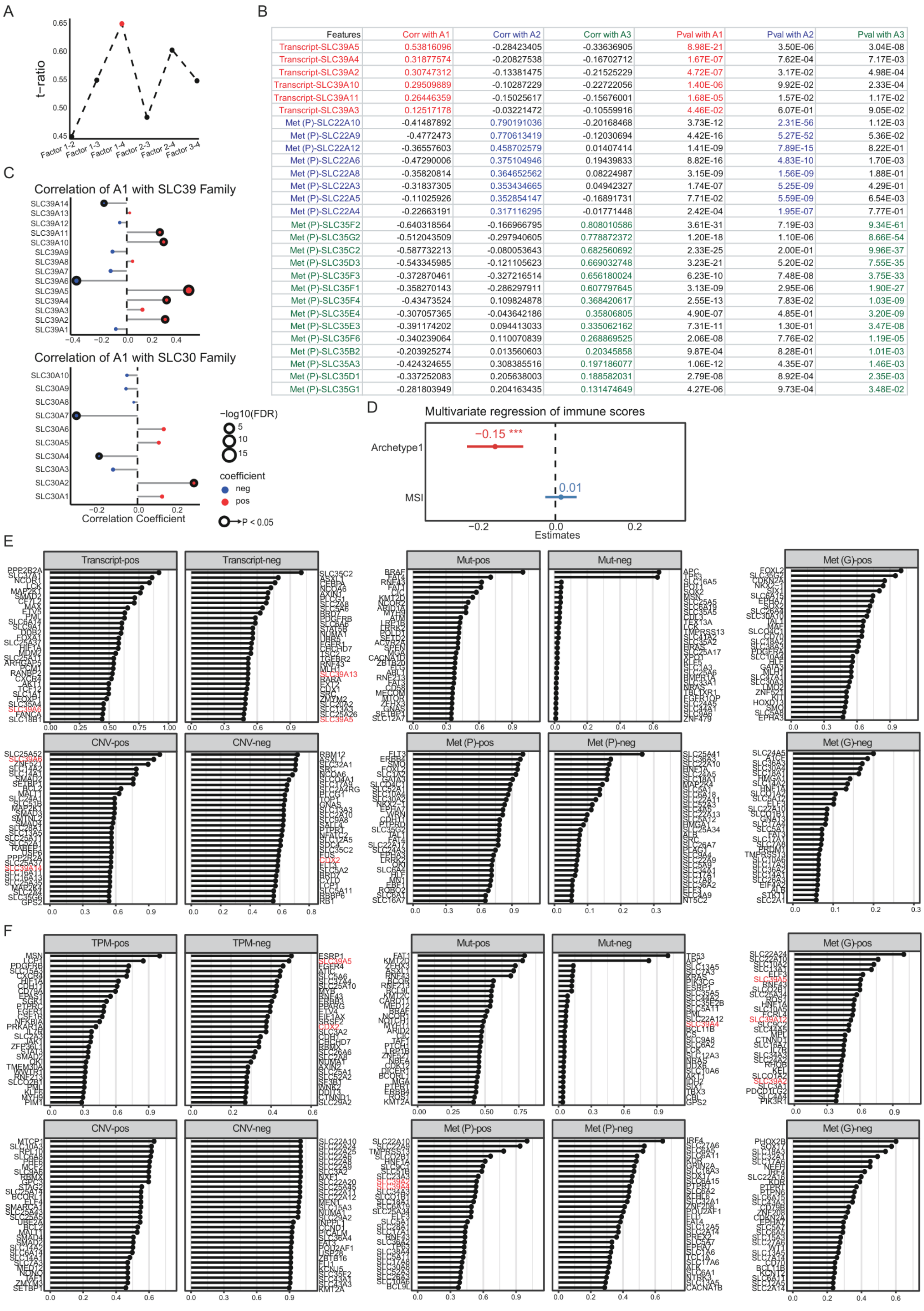

Features	Corr with A1	Corr with A2	Corr with A3	Pval with A1	Pval with A2	Pval with A3
Transcript-SLC39A5	0.53816096	-0.28423405	-0.33636905	8.98E-21	3.50E-06	3.04E-08
Transcript-SLC39A4	0.31877574	-0.20827538	-0.16702712	1.67E-07	7.62E-04	7.17E-03
Transcript-SLC39A2	0.30747312	-0.13381475	-0.21525229	4.72E-07	3.17E-02	4.98E-04
Transcript-SLC39A10	0.29509889	-0.10287229	-0.22722056	1.40E-06	9.92E-02	2.33E-04
Transcript-SLC39A11	0.26446359	-0.15025617	-0.15676001	1.68E-05	1.57E-02	1.17E-02
Transcript-SLC39A3	0.12517178	-0.03221472	-0.10559916	4.46E-02	6.07E-01	9.05E-02
Met (P)-SLC22A10	-0.41487892	0.790191036	-0.20168468	3.73E-12	2.31E-56	1.12E-03
Met (P)-SLC22A9	-0.4772473	0.770613419	-0.12030694	4.42E-16	5.27E-52	5.36E-02
Met (P)-SLC22A12	-0.36557603	0.458702579	0.01407414	1.41E-09	7.89E-15	8.22E-01
Met (P)-SLC22A6	-0.47290006	0.375104946	0.19439833	8.82E-16	4.83E-10	1.70E-03
Met (P)-SLC22A8	-0.35820814	0.364652562	0.08224987	3.15E-09	1.56E-09	1.88E-01
Met (P)-SLC22A3	-0.31837305	0.353434665	0.04942327	1.74E-07	5.25E-09	4.29E-01
Met (P)-SLC22A5	-0.11025926	0.352854147	-0.16891731	7.71E-02	5.59E-09	6.54E-03
Met (P)-SLC22A4	-0.22663191	0.317116295	-0.01771448	2.42E-04	1.95E-07	7.77E-01
Met (P)-SLC35F2	-0.640318564	-0.166966795	0.808010586	3.61E-31	7.19E-03	9.34E-61
Met (P)-SLC35G2	-0.512043509	-0.297940605	0.778872372	1.20E-18	1.10E-06	8.66E-54
Met (P)-SLC35C2	-0.587732213	-0.080053643	0.682560692	2.33E-25	2.00E-01	9.96E-37
Met (P)-SLC35D3	-0.543345985	-0.121105623	0.669032748	3.23E-21	5.20E-02	7.55E-35
Met (P)-SLC35F3	-0.372870461	-0.327216514	0.656180024	6.23E-10	7.48E-08	3.75E-33
Met (P)-SLC35F1	-0.358270143	-0.286297911	0.607797645	3.13E-09	2.95E-06	1.90E-27
Met (P)-SLC35F4	-0.43473524	0.109824878	0.368420617	2.55E-13	7.83E-02	1.03E-09
Met (P)-SLC35E4	-0.307057365	-0.043642186	0.35806805	4.90E-07	4.85E-01	3.20E-09
Met (P)-SLC35E3	-0.391174202	0.094413033	0.335062162	7.31E-11	1.30E-01	3.47E-08
Met (P)-SLC35F6	-0.340239064	0.110070839	0.268869525	2.06E-08	7.76E-02	1.19E-05
Met (P)-SLC35B2	-0.203925274	0.013560603	0.20345858	9.87E-04	8.28E-01	1.01E-03
Met (P)-SLC35A3	-0.424324655	0.308385516	0.197186077	1.06E-12	4.35E-07	1.46E-03
Met (P)-SLC35D1	-0.337252083	0.205638003	0.188582031	2.79E-08	8.92E-04	2.35E-03
Met (P)-SLC35G1	-0.281803949	0.204163435	0.131474649	4.27E-06	9.73E-04	3.48E-02

Fig. S1. Identification of omics features defining CRC archetypes across MOFA dimensions.

(A) T-ratio of polytopes by combination of two MOFA latent factors. **(B)** Correlation analysis between colorectal cancer (CRC) archetype assignment (A1–A3) and individual omics features derived from SLC family genes, including mRNA expression and methylation. Pearson correlation coefficients and corresponding P-values are shown for each archetype. Features with significant positive correlations were color-coded according to their associated archetype (A1: red, A2: blue, A3: green). Enriched omics features derived from SLC family genes are listed below for each archetype, in descending order of correlation strength. **(C)** Transcriptomic profiles of SLC39 (zinc influx) and SLC30A (zinc efflux) family genes were correlated with Archetype 1 enrichment scores across TCGA CRC samples. Multiple membrane-localized SLC39 genes exhibited significant positive correlations with Archetype 1 scores (Spearman correlation; FDR < 0.05), whereas most SLC30A family members showed either no significant association or negative correlations. Notably, SLC30A1, a major membrane zinc efflux transporter, did not show a significant correlation. **(D)** Multivariate linear regression analysis to evaluate whether the negative correlation between Archetype1 scores and immune scores is independent of MSI status. **(E)** Feature-wise contribution scores for MOFA-inferred Factor 1, representing one of the latent dimensions obtained from multi-omics integration. Top-ranked features across omics types (TPM, CNV, mutation, promoter methylation, body methylation) are shown. Members of the SLC39 family and CDX2 are highlighted in red. **(F)** Feature-wise contribution scores for MOFA-inferred Factor 4, visualized as in (E). Top-ranked omics features were identified based on absolute contribution magnitude. Genes highlighted in red denote members of the SLC39 family and CDX2.

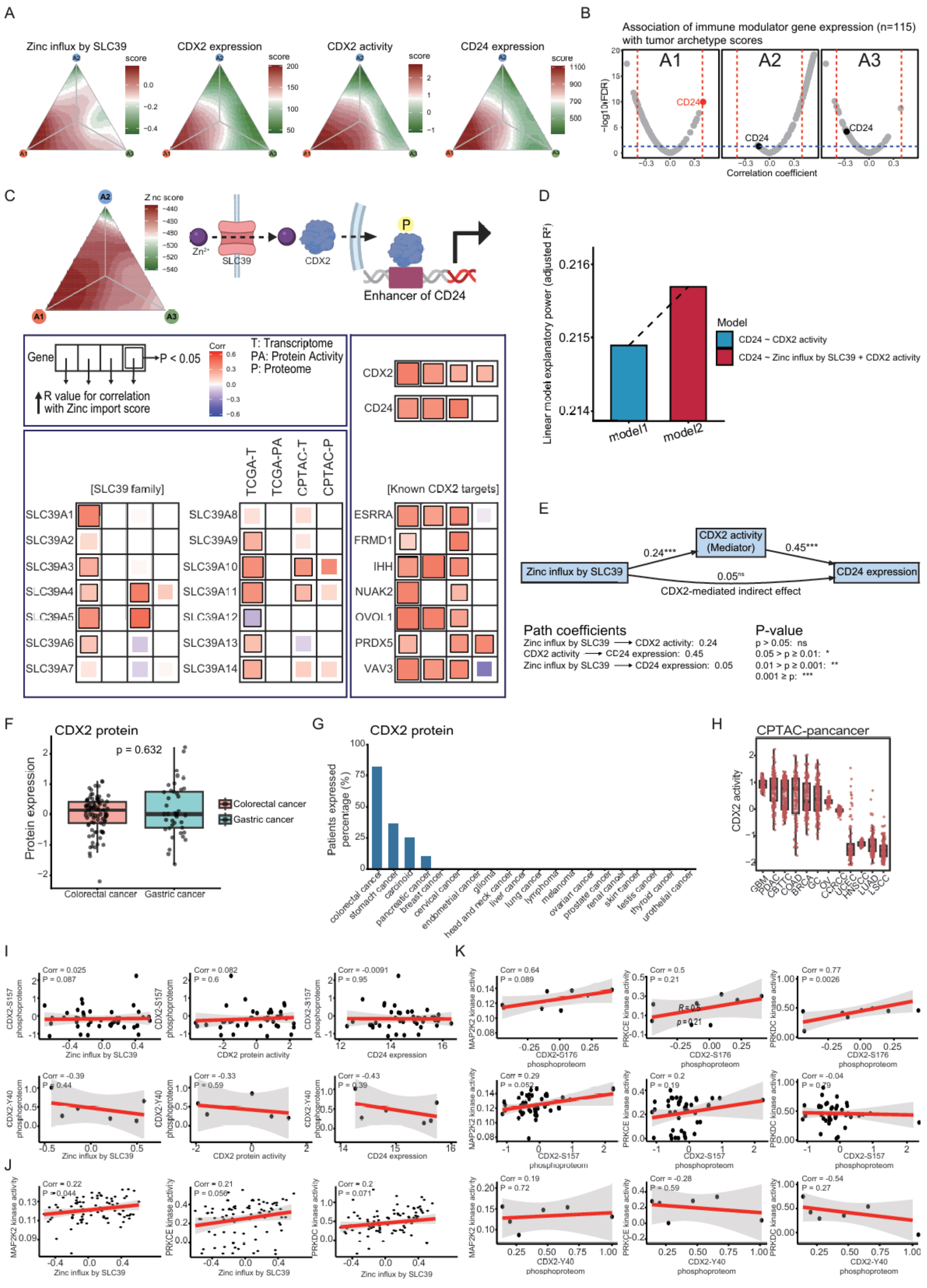

Fig. S2. Integrative assessment of zinc-related transcriptional programs and epigenomic mapping in CRC.

(A) Ternary plots showing the enrichment of molecular features across archetypes A1–A3 derived from Pareto-based archetype inference in TCGA CRC cohort. Four features were visualized: zinc influx by SLC39 scores, CDX2 expression, CDX2 activity, and CD24 expression. Each plot represents score distribution across the triangular archetypal space, with values interpolated across sample coordinates. **(B)** Correlation analysis between CRC archetype assignment (A1–A3) and curated immune modulator gene expression. A total of 115 immune modulator genes were curated from previous reports. Pearson correlation coefficients and corresponding false discovery rates (FDR) are shown for each archetype. CD24 was identified as a top-ranked positively correlated immune modulator gene in Archetype 1, whereas no significant association was observed in Archetype 2 or Archetype 3. **(C)** Integrative correlation analysis of the SLC39–CDX2–CD24 axis across transcriptome and proteome datasets. Correlation matrices were generated using gene expression data from 288 TCGA samples and proteomic data from 90 CPTAC samples to assess the relationships between zinc flux scores—representing extracellular to intracellular zinc import—and the expression of SLC39 family genes, CDX2 activity, and downstream targets. Zinc flux scores were independently computed for each dataset using the COMPASS algorithm. **(D)** Multivariate linear regression models evaluating the contribution of zinc influx by SLC39 and CDX2 activity to CD24 expression. Model fit was compared between the model with and without zinc influx by SLC39 using the adjusted coefficient of determination (adjusted R^2) to account for the difference in the number of predictors. **(E)** Mediation analysis evaluating the relationship between zinc influx by SLC39, CDX2 activity, and CD24 expression in integrated TCGA and CPTAC datasets, with CDX2 activity modeled as a mediator between zinc influx and CD24 expression. Standardized regression coefficients are shown. **(F)** Comparison of CDX2 protein abundance between CRC and gastric cancer samples using CPTAC proteomic datasets. **(G)** Frequency of CDX2 protein detection across human tumor

tissues using pancancer immunohistochemistry data from The Human Protein Atlas. Percentages represent the proportion of patients annotated as exhibiting detectable CDX2 staining across individual tumor types. **(H)** CDX2 activity across pancancer cohorts estimated from CPTAC transcriptomic datasets using VIPER-based regulatory activity inference. Activity scores are shown for individual tumor types. **(I)** Correlation analysis between phosphorylation levels of alternative CDX2 phosphosites (S157 and Y40) and zinc influx by SLC39 scores, CDX2 activity scores, and CD24 expression levels across CPTAC CRC samples. Pearson correlation coefficients and corresponding *P*-values are shown. **(J)** Kinase activities were estimated from phosphoproteomic data of CRC samples in the CPTAC (n = 84) cohort using the KSTAR algorithm. Among kinases previously reported to exhibit zinc-responsive activity, MAP2K2, PRKCE, and PRKDC activity scores showed positive correlations with zinc influx by SLC39. **(K)** Correlation analysis between kinase activity scores (MAP2K2, PRKCE, PRKDC) and phosphorylation levels of CDX2 S176, S157 and Y40 phosphosites in CPTAC CRC samples. Created in BioRender. Lab, S. (2026) https://BioRender.com/6c6ih16.

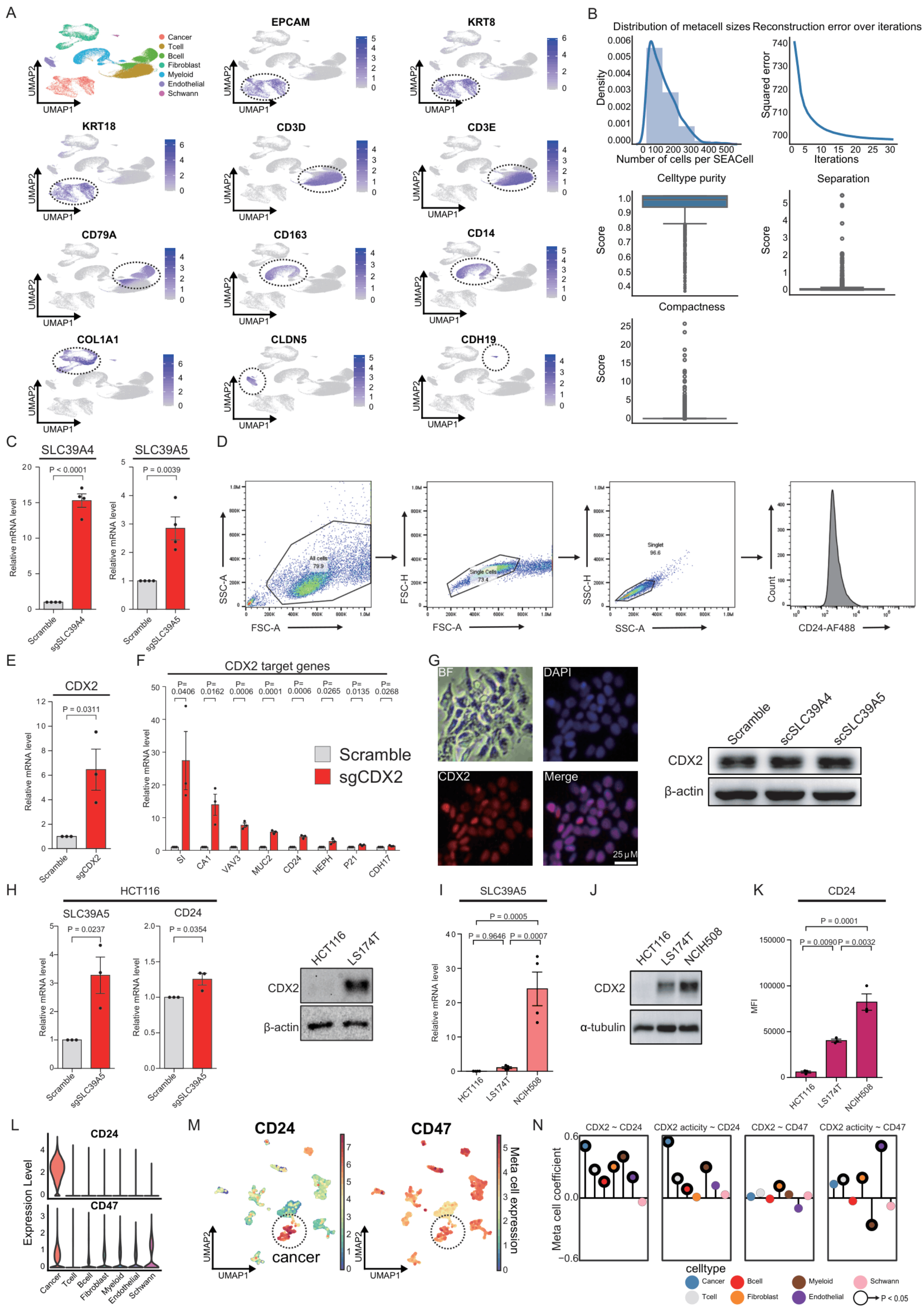

Fig. S3. Metacell construction and evaluation of CD24 and CD47 expression.

(A) Single-cell RNA-seq–based cell type annotation using canonical marker gene expression. Cell types were annotated based on marker gene expression of epithelial (*EPCAM*, *KRT8*, *KRT18*), T cell (*CD3D*, *CD3E*), B cell (*CD79A*), myeloid (*CD163*, *CD14*), fibroblast (*COL1A1*), endothelial (*CLDN5*), and plasma B cell (*CD19*) lineages. Marker gene expression was visualized on UMAP embedding of the reference dataset used in downstream analysis. **(B)** Quality assessment of metacell decomposition using SEACell. Distribution of single-cell counts per metacell and convergence of reconstruction error over optimization iterations (left). Metacell purity calculated as the fraction of dominant cell type in each metacell, and separation and compactness metrics computed from the first 10 diffusion components to evaluate distinctness and internal cohesion of each metacell (right). **(C)** Quantitative RT-PCR analysis of SLC39A4 or SLC39A5 in SLC39A4- or SLC39A5-overexpressing LS174T cells, respectively, confirming overexpression (*n* = 4). **(D)** Gating strategy used in flow cytometry for membrane-associated CD24 fluorescence analysis. **(E** and **F)** Quantitative RT-PCR analysis of CDX2 in CDX2-overexpressing LS174T cells (E) and validation of known target gene expression (F) (*n* = 3). **(G)** Immunofluorescent images of LS174T cells showing nuclear localization of CDX2 protein (left) and Western blot of CDX2 total protein levels in SLC39A4- and SLC39A5-overexpressing cells (right). **(H)** Quantitative RT-PCR analysis of CD24 in SLC39A5-overexpressing HCT116 cells (left). Western blot analysis of CDX2 in HCT116 and LS174T cell line (right) (*n* = 3). **(I)** Expression analysis of the SLC39A5 transcript levels in the non-CMS2 HCT116 and LS174T cells, and CMS2 NCIH508 cells (*n* = 4). (**J**) Expression analysis of the CDX2 protein levels in the non-CMS2 HCT116 and LS174T cells, and CMS2 NCIH508 cells**.** **(K)** Expression analysis of the CD24 membrane localization in the non-CMS2 HCT116 and LS174T cells, and CMS2 NCIH508 cells (*n* = 3). **(L)** Single-cell level expression distribution of CD24 and CD47 across major annotated cell types. **(M)** CD24 and CD47 expression at the metacell level projected onto the

UMAP. Metacell-level expression was computed as the average gene expression across constituent single cells within each metacell. **(N)** Correlation coefficients between metacell-level expression of CD24 or CD47 and regulatory features including CDX2 expression and CDX2 activity. Correlations were computed in a cell type–specific manner, and significance ($P < 0.05$) is denoted by circle fill color. In (C), (E), (F), (H), (I), and (K), data are presented as mean ± SEM. Unpaired two-tailed t-test was used for (C), (E), (F) and (H). One-way ANOVA followed by Tukey's multiple comparisons test was used for (I) and (K).

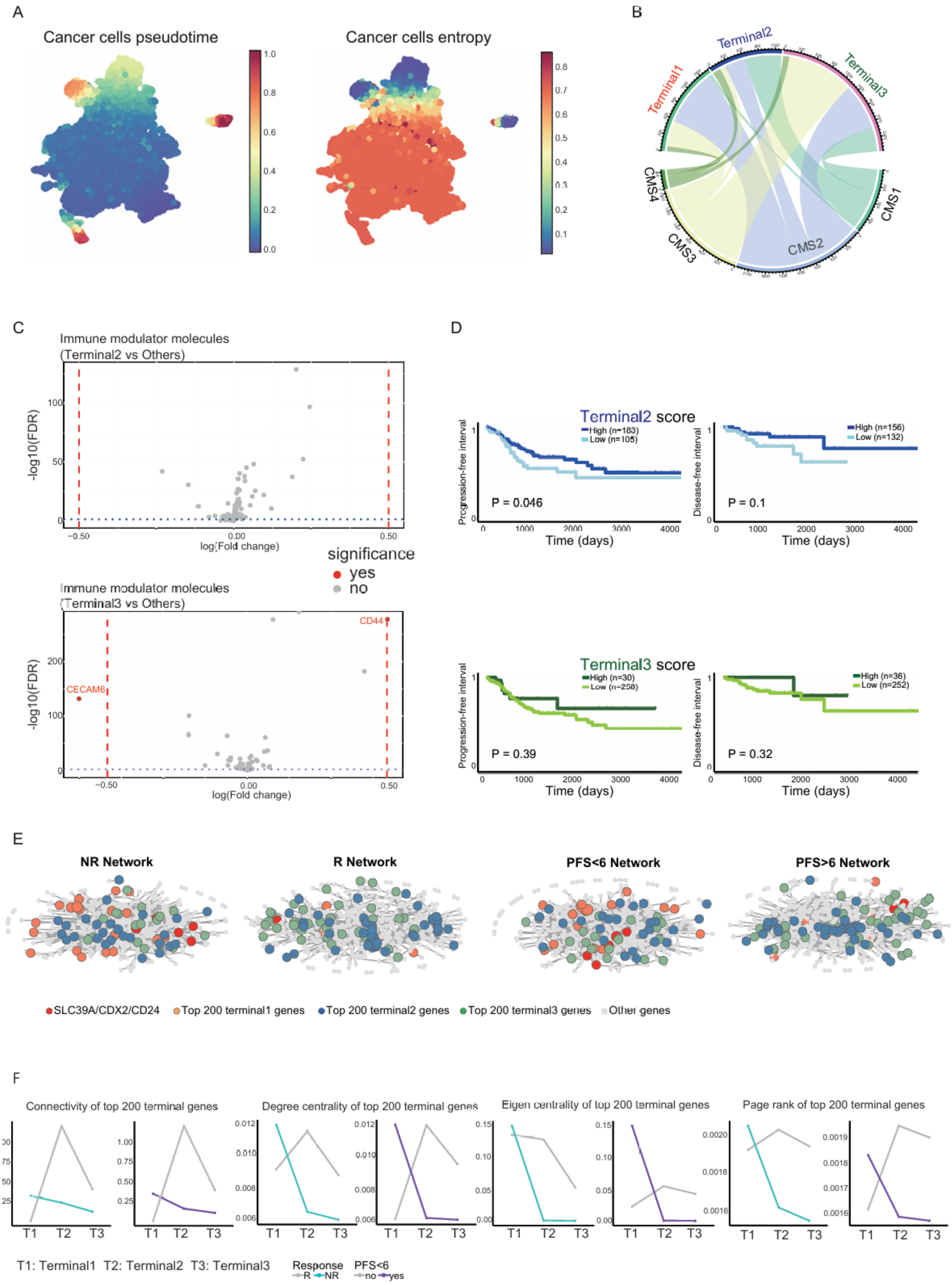

Fig. S4. Trajectory-based characterization of Terminal 2/3 states and network reproducibility metrics.

(A) Pseudotime and entropy values of cancer cells inferred by Palantir, visualized on the UMAP space. Pseudotime reflects progression along the inferred trajectory, while entropy indicates lineage plasticity or transition uncertainty. **(B)** Circular plot showing association between Terminal 1–3 states (defined from pseudotemporal analysis) and consensus molecular subtypes (CMS1–CMS4), based on frequency of CMS label assignment within each terminal cluster. **(C)** Volcano plots displaying differential expression of immune modulatory genes between Terminal 2 vs. others (top) and Terminal 3 vs. others (bottom), using $\log_2$ fold change and $-\log_{10}$(FDR) as axes. Genes surpassing significance thresholds were highlighted. **(D)** Kaplan–Meier plots showing progression-free interval (PFI) and disease-free interval (DFI) stratified by high vs. low scores of Terminal 2 (top) and Terminal 3 (bottom) gene programs. Stratification was based on median score threshold in TCGA-COAD tumors. **(E)** Cell type–specific gene networks were constructed using scHumannet for ICI non-responder (NR), responder (R), PFS < 6 months, and PFS ≥ 6 months groups. Each network was annotated with top 200 terminal gene sets (T1–T3). Genes in the SLC39/CDX2/CD24 axis were marked in red. **(F)** Quantitative network analyses showing centrality metrics of top 200 terminal state–specific genes across each condition. Metrics included connectivity, degree centrality, eigenvector centrality, and PageRank, highlighting the distribution of structural importance across the T1–T3 programs in NR/R and PFS subgroups.

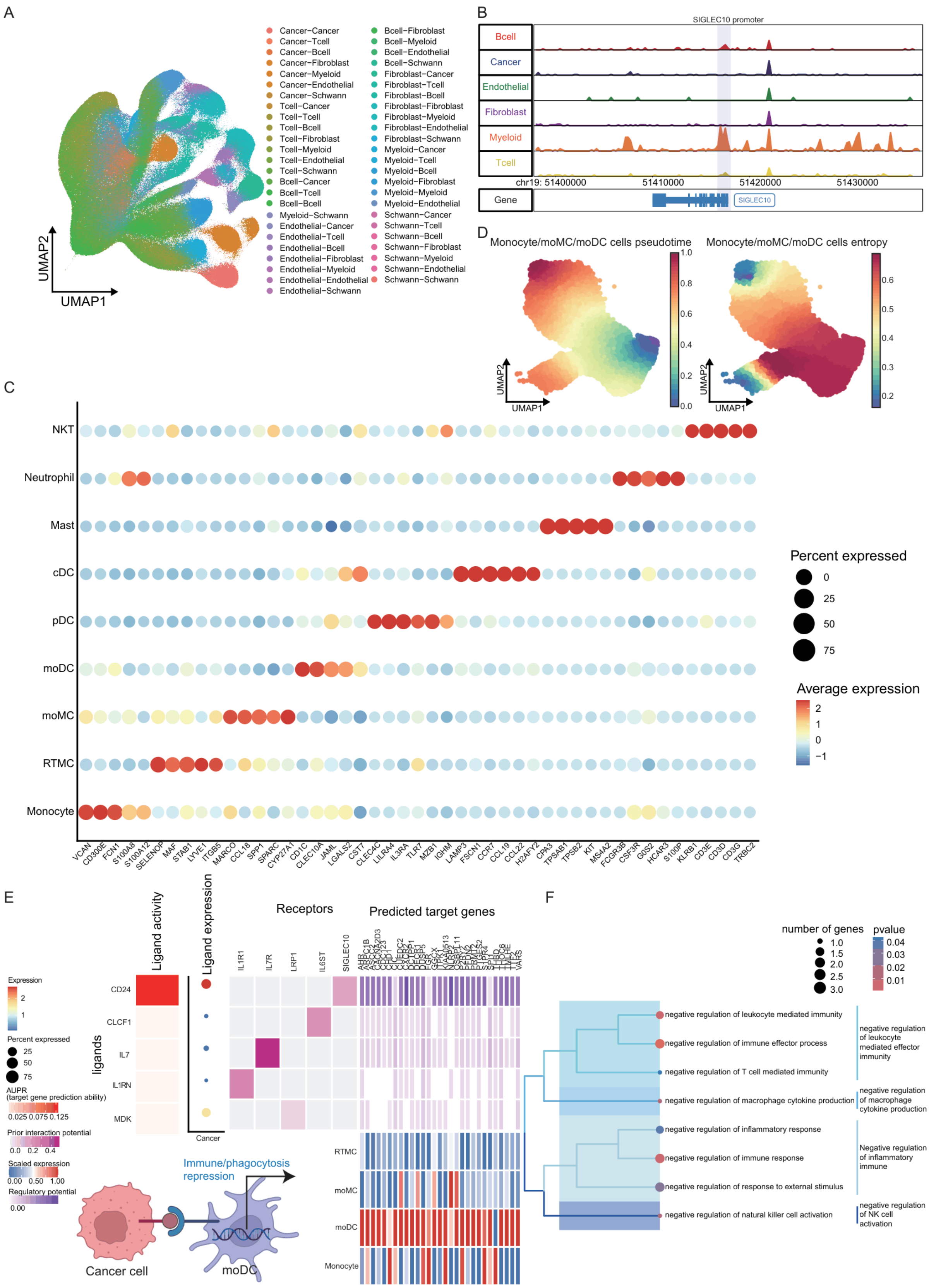
A
UMAP2
UMAP1
Cancer−Cancer
Cancer−Tcell
Cancer−Bcell
Cancer−Fibroblast
Cancer−Myeloid
Cancer−Endothelial
Cancer−Schwann
Tcell−Cancer
Tcell−Tcell
Tcell−Bcell
Tcell−Fibroblast
Tcell−Myeloid
Tcell−Endothelial
Tcell−Schwann
Bcell−Cancer
Bcell−Tcell
Bcell−Bcell
Myeloid−Schwann
Endothelial−Cancer
Endothelial−Tcell
Endothelial−Bcell
Endothelial−Fibroblast
Endothelial−Myeloid
Endothelial−Endothelial
Endothelial−Schwann
Bcell−Fibroblast
Bcell−Myeloid
Bcell−Endothelial
Bcell−Schwann
Fibroblast−Cancer
Fibroblast−Tcell
Fibroblast−Bcell
Fibroblast−Fibroblast
Fibroblast−Myeloid
Fibroblast−Endothelial
Fibroblast−Schwann
Myeloid−Cancer
Myeloid−Tcell
Myeloid−Bcell
Myeloid−Fibroblast
Myeloid−Myeloid
Myeloid−Endothelial
Schwann−Cancer
Schwann−Tcell
Schwann−Bcell
Schwann−Fibroblast
Schwann−Myeloid
Schwann−Endothelial
Schwann−Schwann
B
SIGLEC10 promoter
Bcell
Cancer
Endothelial
Fibroblast
Myeloid
Tcell
chr19: 51400000
51410000
51420000
51430000
Gene
SIGLEC10
D
Monocyte/moMC/moDC cells pseudotime
Monocyte/moMC/moDC cells entropy
UMAP2
UMAP1
1.0
0.8
0.6
0.4
0.2
0.0
0.6
0.5
0.4
0.3
0.2
C
NKT
Neutrophil
Mast
cDC
pDC
moDC
moMC
RTMC
Monocyte
VCAN
CD300E
FCN1
S100A8
S100A12
SELENOP
MAF
STAB1
LYVE1
ITGB5
MARCO
CCL18
SPP1
SPARC
CYP27A1
CD1C
CLEC10A
JAML
LGALS2
CST7
CLEC4C
LILRA4
IL3RA
TLR7
MZB1
IGHM
LAMP3
FSCN1
CCR7
CCL19
CCL22
H2AFY2
CPA3
TPSAB1
TPSB2
KIT
MS4A2
FCGR3B
CSF3R
G0S2
HCAR3
S100P
KLRB1
CD3E
CD3D
CD3G
TRBC2
Percent expressed
0
25
50
75
Average expression
2
1
0
−1
E
Ligand activity
Ligand expression
Receptors
Predicted target genes
Expression
2
1
Percent expressed
25
50
75
AUPR
(target gene prediction ability)
0.025 0.075 0.125
Prior interaction potential
0.0 0.2 0.4
Scaled expression
0.00 0.50 1.00
Regulatory potential
0.00
ligands
CD24
CLCF1
IL7
IL1RN
MDK
Cancer
IL1R1
IL7R
LRP1
IL6ST
SIGLEC10
RTMC
moMC
moDC
Monocyte
Immune/phagocytosis
repression
Cancer cell
moDC
F
number of genes
1.0
1.5
2.0
2.5
3.0
pvalue
0.04
0.03
0.02
0.01
negative regulation of leukocyte mediated immunity
negative regulation of immune effector process
negative regulation of T cell mediated immunity
negative regulation of macrophage cytokine production
negative regulation of inflammatory response
negative regulation of immune response
negative regulation of response to external stimulus
negative regulation of natural killer cell activation
negative regulation of leukocyte mediated effector immunity
negative regulation of macrophage cytokine production
Negative regulation of inflammatory immune
negative regulation of NK cell activation

Fig. S5. Multi-modal validation of cancer–myeloid CD24–SIGLEC10 interaction and myeloid subtype development.

(A) Sender–receiver interaction embeddings across cell pairs in scRNA-seq data (same dataset as used in Fig. 3), visualized using NICHES. Each dot represents a sender–receiver cell pair, and colors correspond to combinations of sender and receiver identities. **(B)** Chromatin accessibility peaks at the *SIGLEC10* promoter locus (shaded region) across six major cell types, computed as the mean from three CRC scATAC-seq samples. Notably, specific peaks are observed in the myeloid lineage. **(C)** Dot plot showing expression of marker genes across annotated myeloid subtypes including Monocyte, moDC, moMC, RTMC, cDC, and pDC. Dot size reflects the percentage of expressing cells, and color indicates average expression level. **(D)** Trajectory inference using diffusion-based pseudotime and entropy mapping of myeloid subtypes. Pseudotime was inferred from Monocytes toward moMC or moDC terminal fates, with entropy quantifying transcriptional heterogeneity along each branch. **(E)** Ligand–receptor analysis using NicheNet focused on cancer–moDC interactions. Left: Heatmaps show ligand activity scores and expression of candidate ligands in cancer cells. Middle: Receptor usage and predicted downstream target genes in moDCs are shown. Bottom-left: Schematic illustrating signaling from cancer to moDCs. Right: Heatmap displays predicted downstream genes ranked by regulatory potential. **(F)** Functional enrichment analysis of the predicted target genes identified in moDCs (from panel E) using Gene Ontology Biological Process terms. Dot size reflects the number of enriched genes and color indicates the enrichment p-value. Created in BioRender. Lab, S. (2026) https://BioRender.com/qcu9zcs.

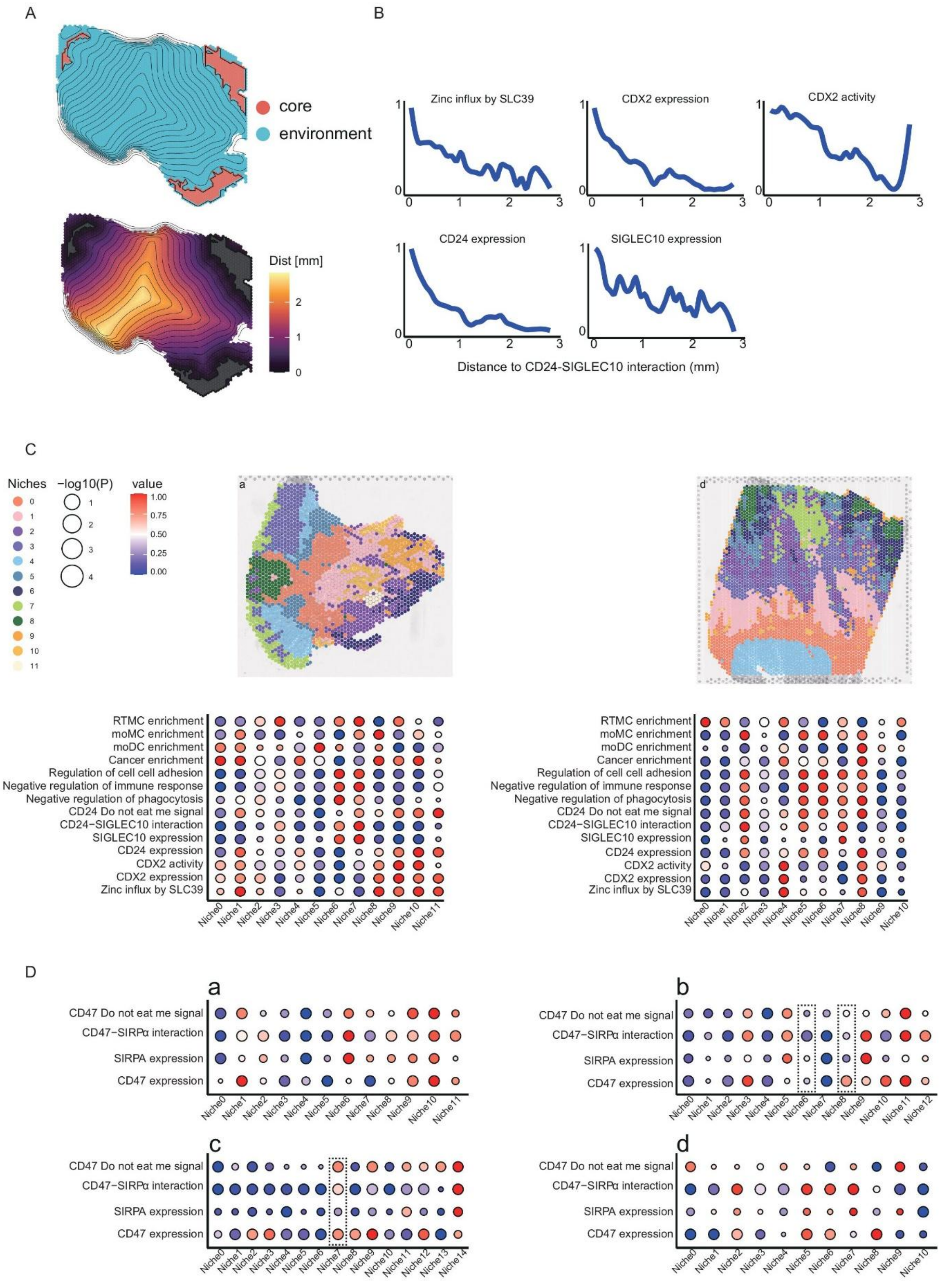

Fig. S6. Robust spatial characterization of immune-modulatory niches enriched for CD24–SIGLEC10 and CD47–SIRPα axes across CRC samples.

(A) Spatial domain annotation using SPATA2 highlighting CD24–SIGLEC10 interaction-enriched regions ("core") and surrounding regions ("environment") across CRC spatial transcriptomic datasets (*n* = 4 samples). The bottom panel shows distance transform (in mm) from the enriched region boundary, used for local spatial expression modeling. Screening direction was computed using ggpLayerScreeningDirectionSAS() in SPATA2. **(B)** Distance-based spatial modeling of zinc influx by SLC39, CDX2 expression, CDX2 activity, CD24 expression and SIGLEC10 expression across cancer cell–enriched regions. Loess-fitted expression curves demonstrate a gradient of decreasing zinc–CD24–SIGLEC10 axis activity from the enriched core toward the periphery. **(C)** Additional spatial samples (Samples 1 and 4), labeled as subimages (a) and (d), respectively, with lower enrichment of zinc–CD24–SIGLEC10 signaling were profiled for local enrichment of pathway signatures, cell types (RTMC, moMC, and moDC), and key molecules (CDX2, CD24, and SIGLEC10), showing sample-specific spatial variation. **(D)** Spatial enrichment patterns of the CD47–SIRPα axis across all four CRC samples. Dot plots indicate gene set enrichment scores and CD47–SIRPα interaction strength across annotated spatial niches. Notably, boxed regions in Samples 2 and 3, labeled as subimages (b) and (c), respectively, exhibited consistent co-enrichment of CD24–SIGLEC10 signaling and RTMC/moMC infiltration, whereas CD47–SIRPα signaling remained inactive, highlighting selective engagement of the CD24–SIGLEC10 axis within immune-suppressive niches.

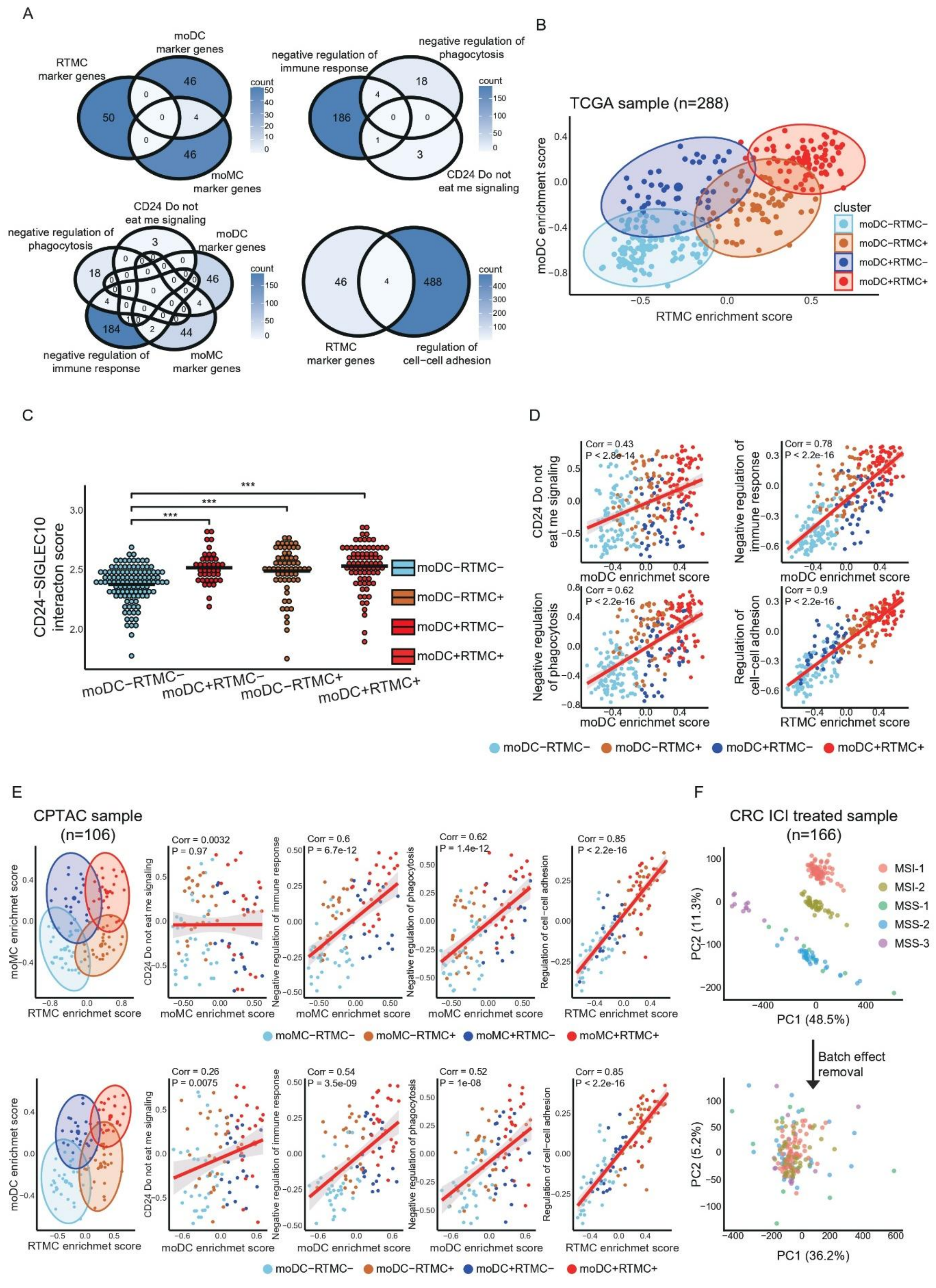

Fig. S7. Validation of immunosuppressive axis across moDC–RTMC programs and CPTAC dataset.

(A) Venn diagrams showing overlap between cell type–specific marker gene sets (moMC, moDC, RTMC; 50 genes per population) and four relevant immune-related gene sets: CD24 “don’t eat me” signaling, negative regulation of phagocytosis, negative regulation of immune response, and regulation of cell–cell adhesion. Each plot displays pairwise intersection between a given marker set and one pathway gene set. **(B)** Marker gene sets (*n* = 50 per population) from single-cell–defined moDC and RTMC subtypes were used to score bulk RNA-seq profiles from the TCGA-COAD cohort (*n* = 288). Scores were plotted in 2D and clustered using fviz_cluster() from the factoextra package, yielding four distinct subgroups based on moDC and RTMC axis scores. **(C)** CD24–SIGLEC10 interaction scores were inferred from TCGA bulk expression data using DecoupleR, applying ligand–receptor pair sets from the LIANA database. Each pair was treated as a gene set, and enrichment scores were computed using weighted mean-based scoring. Interaction scores were compared across the four moDC/RTMC-defined subtypes. **(D)** Bulk-level correlation analysis between moDC and RTMC axis scores and pathway activities. moDC scores were evaluated against CD24-mediated “don’t eat me” signaling, negative regulation of phagocytosis, and negative regulation of immune responses. RTMC scores were assessed for correlation with the regulation of cell–cell adhesion. **(E)** Reproduction of analyses shown in Fig. 7A and Fig. 7C using bulk RNA-seq data from the CPTAC-COAD cohort (*n* = 106). Top row: axis scoring and subtype clustering using moMC and RTMC marker gene sets. Bottom row: same analysis using moDC and RTMC marker sets. **(F)** Principal component analysis (PCA) before and after batch correction of ICI-treated CRC bulk RNA-seq cohort. Samples from five integrated cohorts (MSS1, MSS2, MSS3, MSI1, MSI2; *n* = 166) were integrated and visualized using PCA. Top, uncorrected gene expression data show distinct clustering by batch. Bottom, batch effects were removed using the removeBatchEffect() function from the limma package, followed by PCA. Color indicates cohort origin.

Supplementary Table S1. Clinical data for TCGA cohort.

Supplementary Table S2. Clinical data for CPTAC cohort.

Supplementary Table S3. Clinical data for scATAC-seq cohort.

Supplementary Table S4. Clinical data for scRNA-seq.

Supplementary Table S5. Clinical data for ICI treated scRNA-seq cohort.

Supplementary Table S6. Clinical data for 10x Genomics Visium cohort.

Supplementary Table S7. Primer sequences for RT-qPCR.

Supplementary Table S8. Clinical data for ICI treated bulk RNA-seq cohort.

Supplementary Table S9. Summary of immune checkpoint inhibitor response biomarkers used for benchmarking.

Data S1. Raw data for Figures 3 and S3 used to generate the corresponding graphs.

Data S2. Raw data for Figures 7 to generate the corresponding graphs.
